# CuS-Bridged MXene-Based Photoresponsive Phase Change Materials Enabling Thermoelectric Cogeneration and Microwave Absorption

**DOI:** 10.1007/s40820-026-02277-7

**Published:** 2026-07-07

**Authors:** Yuhao Feng, Jindi Zhao, Keke Chen, Yang Li, Jiao Liu, Huitao Yu, Mulin Qin, Kaihang Jia, Haiwei Han, Zhenghui Shen, Mingliang Ma, Xiao Chen

**Affiliations:** 1 School of Physics and Astronomy, Beijing Normal University, Beijing, 100875 People’s Republic of China; 2 School of Materials Science and Engineering, Peking University, Beijing, 100871 People’s Republic of China; 3 School of Civil Engineering, Qingdao University of Technology, Qingdao, 266033 People’s Republic of China; 4 School of Materials Science and Engineering, Inner Mongolia University of Science and Technology, Baotou, 014010 Inner Mongolia People’s Republic of China

**Keywords:** Phase change materials, CuS-bridged MXene, Photothermal conversion, Thermoelectric cogeneration, Microwave absorption

## Abstract

Designing multifunctional materials integrating efficient photoresponsive thermoelectric cogeneration with microwave absorption remains challenging for clean solar energy utilization and electromagnetic interference mitigation. Herein, this study proposes an innovative design strategy combining interfacial bridging engineering and physical encapsulation to construct advanced multifunctional composite phase change materials (PCMs). A nanoparticle-bridged 3D interpenetrating phonon network is constructed via in-situ growth of CuS nanoparticles on layered MXene, creating abundant interfacial sites for polyethylene glycol (PEG) adsorption and efficient electron transport channels for electromagnetic loss. The resulting PEG-MXene@CuS composite PCMs achieve an exceptional photothermal conversion efficiency of 94.5% (100 mW cm^−2^), enabled by broadband absorption of MXene nanosheets and plasmonic effect of CuS nanoparticles. In photoresponsive thermoelectric cogeneration system, PEG-MXene@CuS serves as a heat source to drive thermoelectric module through the Seebeck effect, yielding a stable power output of 21.7 W m^−2^ (100 mW cm^−2^), with PEG effectively buffering thermal fluctuations. Moreover, MXene@CuS heterointerface and 3D conductive network endow PEG-MXene@CuS with superior microwave absorption, a minimum reflection loss of − 55.5 dB and an effective bandwidth of 5.27 GHz. This design concept provides important insights into designing next‑generation multifunctional PCMs with promising applications in intelligent thermal management, thermoelectric cogeneration, and electromagnetic protection.

**Supplementary Information:**

The online version contains supplementary material available at 10.1007/s40820-026-02277-7.

## Hightlights

- An interfacial bridging strategy is utilized to construct a 3D interpenetrating network for accelerated phonon/electron transport.
- The synergy of localized surface plasmon resonance effects and phase change thermal buffering enables efficient solar harvesting and stabilized thermoelectric cogeneration.
- Enhanced interfacial polarization and optimized impedance matching significantly boost electromagnetic energy dissipation.

**Supplementary Information:**

The online version contains supplementary material available at 10.1007/s40820-026-02277-7.

## Graphical abstract

**Figure:**
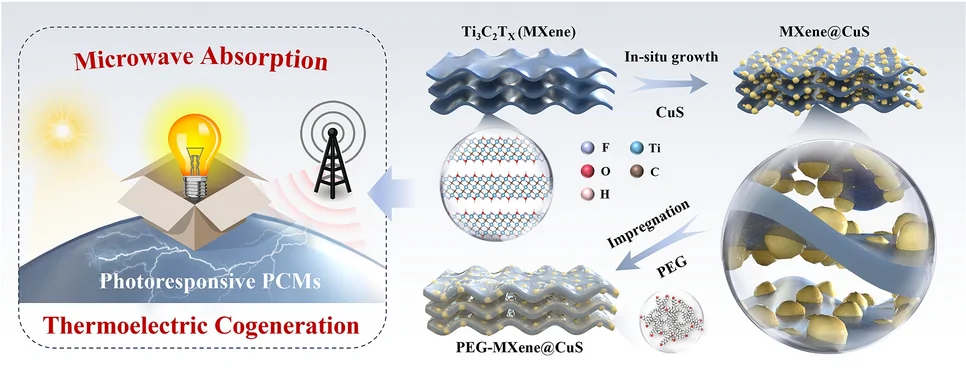

## Introduction

The rapid expansion of high-power electronic devices and increasing demand for sustainable energy have elevated the development of multifunctional systems capable of concurrent energy harvesting and electromagnetic protection to a strategic imperative [1–4]. Conventional materials primarily address energy storage or microwave absorption in isolation, failing to satisfy the sophisticated requirements of next-generation intelligent protective skins for aerospace vehicles and 5G/6G communication infrastructures [5–8]. In these scenarios, a robust energy-protection synergy is essential: the system should function as a photothermal converter to capture solar energy, phase change materials (PCMs) to store latent heat, and thermoelectric generators to power local sensors, while simultaneously maintaining efficient microwave absorption to ensure signal integrity and radar stealth. Consequently, integrating high-efficiency thermal management with reliable electromagnetic attenuation is indispensable for operational stability and autonomy of advanced equipment in extreme environments.

Multifunctional systems generally rely on rational microstructural engineering and multi-component integration to address these challenges. For instance, Sun et al. developed multi-stage anisotropic cellulose-based aerogels through a foam-flake-fiber interweaving strategy, achieving broadband microwave absorption alongside photothermal anti-icing properties [9]. Zheng et al. fabricated scalable MXene/polyurea aerogels enabling synergistic defense against mechanical shocks and electromagnetic interference [10]. Furthermore, Zong et al. constructed hollow MXene@carbon microfiber composites demonstrating efficient light-responsive and microwave absorption [11], while Liang et al. integrated microwave absorption, photothermal, and antimicrobial functions within sea-urchin-like yolk-shell nanostructures [12]. Despite significant strides, achieving high-level electromagnetic attenuation while maintaining high energy-storage capacity remains highly challenging, particularly regarding optimization of internal transport pathways and interfacial impedance.

As a typical two-dimensional (2D) transition metal carbide, Ti_3_C_2_T_x_ MXene has demonstrated immense potential in both energy conversion and electromagnetic attenuation due to its high metallic conductivity and abundant surface chemistry [13–17]. However, intrinsic high reflectivity of pristine MXene layers limits its light-harvesting efficiency, and excessive conductivity induces severe impedance mismatch in microwave absorption applications [18, 19]. To overcome these limitations, semiconductor-based heterostructures, such as MXene/CuS hybrids, have been rationally engineered. For instance, Su et al. reported hierarchical Ti_3_C_2_T_x_/CuS nanofluids where CuS nanoparticles increased surface scattering centers to boost photothermal efficiency [20]. Similarly, Ren et al. demonstrated that in-situ grown CuS can effectively regulate dielectric properties and optimize impedance matching for wide-frequency absorption [21]. From a thermal management perspective, thermal energy storage constitutes a pivotal component in multifunctional systems, where PCMs serve as core functional medium for reversible heat storage and temperature regulation [22–24]. As a promising organic PCM, polyethylene glycol (PEG) possesses high latent heat, excellent chemical stability and reversible phase change behavior, endowing composite systems with reliable thermal buffering and energy-storage efficacy [25, 26]. Nevertheless, several critical challenges persist, including precise control over interfacial coupling to construct efficient cross-interface energy and charge transport pathways, as well as stable encapsulation of PEG within such a heterostructure to achieve mechanical integrity and multifunctional integration.

Herein, a synergistic strategy combining interfacial bridging engineering with physical encapsulation is proposed to construct the multifunctional composite PCMs integrating photothermal conversion, thermoelectric cogeneration, thermal storage, and microwave absorption. By in-situ growing CuS nanoparticles as interlayer pillars on 2D layered MXene, a 3D interpenetrating phonon/electron transport network is established, providing abundant interfacial sites and stable physical confinement for PEG. This unique bridged structure effectively alleviates interfacial thermal resistance and accelerates heat transfer while preventing liquid leakage. The resulting PEG-MXene@CuS composite exhibits broadband solar absorption and superior photothermal conversion capability. In photoresponsive thermoelectric cogeneration system, the composite PCMs serve as a heat source to drive the thermoelectric module via the Seebeck effect, with PEG effectively buffering thermal fluctuations. Furthermore, optimized impedance matching and enhanced interfacial polarization endow the composite PCMs with outstanding microwave absorption. This study provides novel insights and feasible strategies for design of high-performance composite materials with integrated energy regulation and electromagnetic protection.

## Experimental Section

### Materials

Ti_3_AlC_2_ (MAX phase, 98%, 400 mesh) was supplied by 11 Technology Co., Ltd. Copper(II) sulfate pentahydrate (CuSO_4_·5H_2_O) and thiourea (CH_4_N_2_S) were obtained from Shanghai Aladdin Biochemical Technology Co., Ltd. Poly(ethylene glycol) (PEG, Mw ≈ 8000 g mol^−1^) was purchased from Shanghai Macklin Biochemical Co., Ltd. Hydrofluoric acid (HF, 40 wt%) was provided by Innochem Technology Co., Ltd. All the chemicals were analytical grade without further purification.

### Preparation of PEG-MXene@CuS Composite PCMs

#### Preparations of MXene@CuS

Multilayer MXene was first prepared according to a previously reported method [53]. MXene@CuS was subsequently synthesized via in-situ hydrothermal route, where CuS nanoparticles were grown directly on MXene surfaces. In a typical procedure, an amount of MXene was dispersed in 40 mL of deionized water, followed by the addition of equimolar CuSO_4_·5H_2_O and thiourea (CH_4_N_2_S). After magnetic stirring for 30 min to achieve the homogeneous mixture, the solution was transferred into a 100 mL Teflon lined stainless steel autoclave and heated at 180 °C for 24 h. During this process, thiourea decomposition provided the sulfur source, enabling the in-situ formation of CuS nanoparticles anchored onto MXene. After cooling naturally to room temperature, the products were collected, washed thoroughly with deionized water, and freeze dried to obtain the final MXene@CuS powders. Based on the different precursor mass ratios of MXene to CuSO_4_·5H_2_O, the resulting samples were denoted as MXene@CuS-1 (1.5:1), MXene@CuS-2 (1:1), and MXene@CuS-3 (1:2), respectively.

#### Preparations of PEG-MXene@CuS

Composite PCMs were fabricated via the solution impregnation method. Before compounding, the as-prepared MXene@CuS was dried in a vacuum oven at 80 °C for 24 h to remove residual moisture from its porous structure. PEG and MXene@CuS were then dispersed in ethanol at a fixed mass ratio of 7:3. The mixture was vigorously stirred until the ethanol completely evaporated. Subsequently, the resulting samples were placed on filter paper and heated at 80 °C to remove any excess PEG on the surface, until no further leakage occurred, yielding a homogeneous PEG-MXene@CuS composite. For comparison, three composites were prepared following the same procedure, PEG-MXene@CuS-1, PEG-MXene@CuS-2, and PEG-MXene@CuS-3, respectively.

### Characterizations

#### Structural and Chemical Characterizations

The morphology and structure were analyzed using scanning electron microscope (SEM, Regulus 8100) and a transmission electron microscope (TEM, FEI TF200). The crystalline structure and phase composition were identified via X-ray diffraction (XRD, Bruker D8 Advance) with Cu Kα radiation (λ = 1.5406 Å) and Raman spectroscopy (Horiba Hr Evolution). The functional groups and chemical bonding were evaluated through Fourier-transform infrared spectroscopy (FT-IR, Thermo Scientific Nicolet iS20) in 400–4000 cm^−1^. The surface chemical state and elemental composition were determined via X-ray photoelectron spectroscopy (XPS, AXIS Supra) using Al Kα excitation.

#### Thermal Property Characterizations

Phase change temperatures and enthalpies were measured by differential scanning calorimetry (DSC, TA Instruments Q200) at a heating/cooling rate of 10 °C min^−1^ under a nitrogen atmosphere. Thermal stability was evaluated by thermogravimetric analysis (TGA, Mettler Toledo TG/DTA) from room temperature to 800 °C at a heating rate of 10 °C min^−1^ under N_2_ flow. The thermal conductivity was measured using a laser flash analyzer (LFA, Netzsch LFA 467 HyperFlash) at 25 °C.

#### Photothermal and Photothermoelectric Tests

**Photothermal Tests:** The light absorption properties across a broad spectrum were evaluated using UV–Visible-Near Infrared (UV–Vis-NIR) spectroscopy (Agilent Cary 5000). Photothermal conversion performance was assessed under simulated solar irradiation from a xenon lamp (PLS-SXE300D/300DUV, with an AM 1.5G filter). The surface-temperature evolution and distribution were monitored in real-time using an infrared thermal camera (FLIR R2100).

**Photothermoelectric Tests:** The photothermoelectric output was measured using a custom-built device. The composite sample served as the hot side under illumination, while an ice-water bath maintained the cold side. The generated voltage and current signals from the connected commercial thermoelectric generator (TEG) were recorded by an electrochemical workstation (PARSTAT MC).

#### Microwave Absorption Tests

For microwave absorption tests, the PEG-MXene@CuS composite powders were directly pressed into standard toroidal samples (outer diameter: 7.00 mm, inner diameter: 3.04 mm) using a mold and a hydraulic press. The complex permittivity and permeability in the 2–18 GHz frequency range were acquired using a vector network analyzer (Agilent N5222A) via the coaxial line method. The reflection loss (RL) and effective absorption bandwidth (EAB) were calculated based on the measured electromagnetic parameters using the transmission line theory (detailed formulas are provided in the Supporting Information). Far field radar cross-section (RCS) simulations were performed using CST Studio Suite 2024 to evaluate the potential for radar stealth applications.

## Results and Discussion

### Chemical and Structural Analysis

**Fig. 1:**
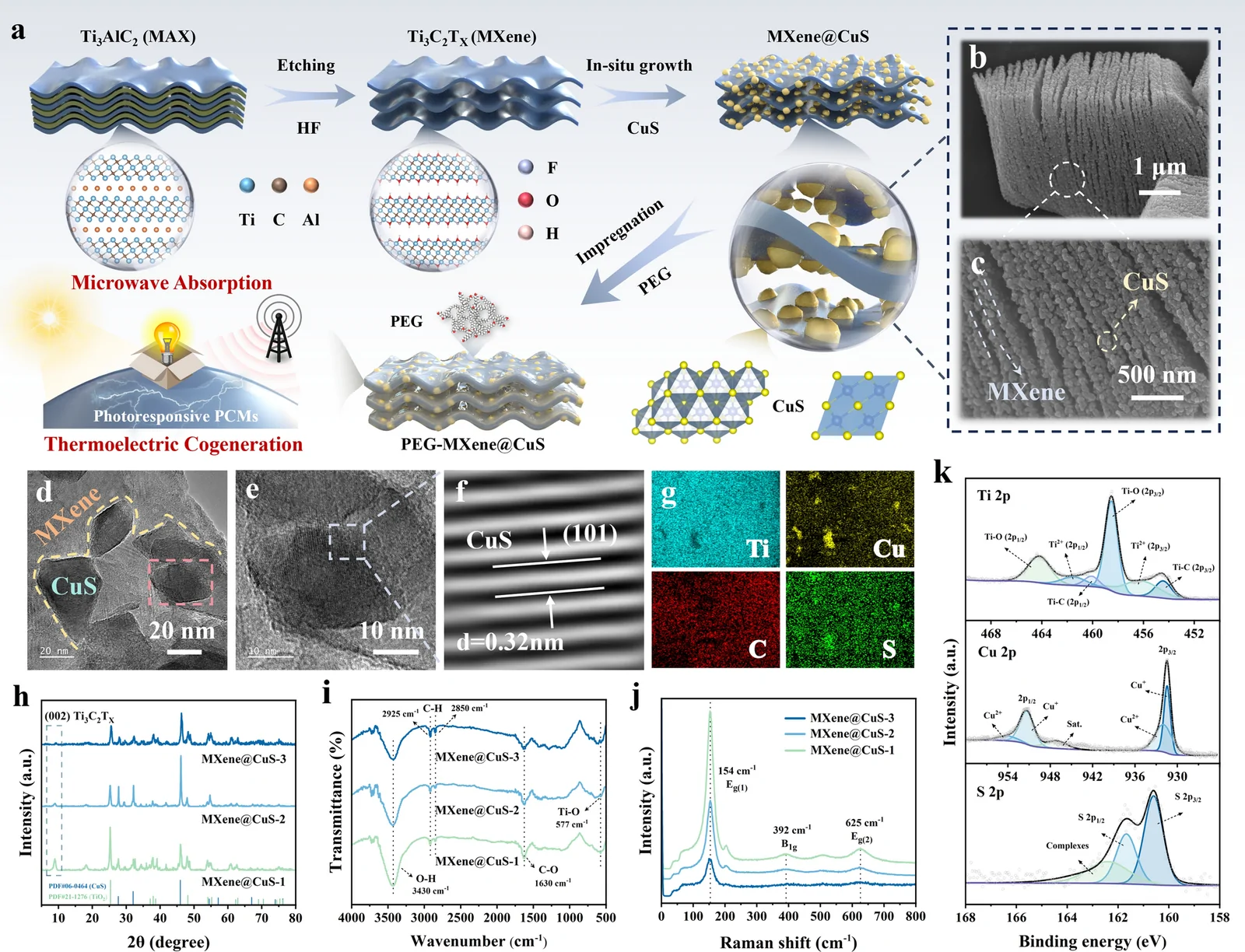
**a** Schematic synthesis illustration of PEG-MXene@CuS composite PCMs **b, c** SEM images of MXene@CuS. **d**–**f** TEM and HRTEM images of MXene@CuS. **g** EDS elemental mapping of MXene@CuS. **h** XRD patterns, **i** FT-IR spectra, **j** Raman spectra, and **k** XPS spectra of MXene@CuS

Figure 1a illustrates the synthesis strategy of CuS-bridged MXene-based composite PCMs for photoresponsive thermoelectric cogeneration and microwave absorption applications. The process begins with the selective etching of Ti_3_AlC_2_ MAX phase precursor using hydrofluoric acid (HF). Owing to the high reactivity of the Al layers, HF reacts with them to form soluble aluminum fluoride and hydrogen gas, thereby chemically selectively etching Al layers and yielding typical accordion-like multilayered MXene (Ti_3_C_2_T_x_) nanosheets with interlayer expansion and wrinkling (Fig. S1). The expanded structure and surface functional groups (e.g., –OH, –F) of MXene nanosheets provide active sites for subsequent Cu^2+^ adsorption (Fig. S2). Subsequently, CuS nanoparticles are grown in-situ on MXene surface via hydrothermal method. Specifically, functional groups such as hydroxyl (–OH) on MXene selectively adsorb Cu^2+^ ions through coordination, creating uniform nucleation sites. Under hydrothermal conditions, the sulfur source decomposes to release S^2−^, which reacts with the adsorbed Cu^2+^ at the interface, initiating the heterogeneous nucleation of CuS. MXene@CuS with different CuS loadings were prepared via tuning the relative precursor concentration of Cu source during the hydrothermal reaction (Figs. S3–S5, Table S1). Significantly, in optimized MXene@CuS-2, MXene framework remains intact while CuS nanoparticles form “bridging” structures (CuS-bridged MXene) that links adjacent MXene layers (Fig. 1b). This CuS-bridged configuration enhances interlayer connectivity and interfacial interactions, ultimately constructing a 3D interconnected heterostructure. CuS nanoparticles (20–200 nm) with hexagonal crystal structure are uniformly distributed on MXene surface and within the interlayer spaces, effectively avoiding agglomeration and thereby facilitating the transport of phonons, photons, and electrons (Fig. 1c).

In contrast, for MXene@CuS-1 with a lower CuS loading, the accordion-like architecture of MXene remains well preserved. However, the attached CuS nanoparticles are sparse and unevenly distributed across the surface and interlayer spaces, failing to form continuous conductive bridge framework. For MXene@CuS-3 with an excessively high CuS loading, severe CuS nanoparticle aggregation is observed in SEM images. Under such conditions, surplus CuS tends to undergo homogeneous nucleation, forming large aggregates rather than uniformly nucleating heterogeneously on MXene, which even partially disrupts the layered architecture of MXene (Figs. S6–S8). TEM further reveals the bridge structure in MXene@CuS-2 (Fig. 1d). CuS nanoparticles show distinct (101) crystal planes with an interplanar spacing of approximately 0.32 nm. The heterogeneous interface between CuS and MXene displays good lattice matching, tight bonding, and no detectable amorphous layer (Fig. 1e, f). Elemental mapping further confirms the uniform distribution of C, Ti, Cu, and S, verifying the homogeneous dispersion of CuS nanoparticles on the MXene scaffold (Fig. 1g). Finally, polyethylene glycol (PEG) was incorporated into MXene@CuS heterostructure via solution impregnation. Driven by capillary forces, PEG molecules infiltrate the interlayer galleries of MXene and the interstices between CuS nanoparticles. Simultaneously, PEG forms hydrogen bonds with MXene@CuS through its ether linkages and terminal hydroxyl groups, resulting in uniform and stable encapsulation. The resulting PEG-MXene@CuS composite PCMs offer a promising platform for efficient photothermalelectric conversion and microwave absorption applications.

XRD was performed on MXene@CuS to systematically investigate the influence of CuS loading on their phase composition and crystal structure (Fig. 1h). All samples exhibit the characteristic diffraction peak (002) of MXene at around 9°, together with distinct diffraction peaks of CuS at 27.8°, 32.2°, and 46.1° (PDF#06–0464), confirming the successful construction of MXene@CuS heterostructures. Notably, additional diffraction peaks appear at 25.2°, 37.8°, and 47.9°, corresponding to the (101), (004), and (200) planes of anatase TiO_2_ (PDF#21–1272), indicating that partial oxidation of Ti_3_C_2_T_x_ MXene occurred during the hydrothermal process. For MXene@CuS-1 with lower CuS loading, the CuS diffraction peaks are relatively weak, suggesting low crystallinity and a limited loading. In contrast, MXene@CuS-2 with moderate CuS loading displays well-defined diffraction features from both MXene and CuS without detectable impurity phases, indicating the formation of well-constructed heterostructure. However, for MXene@CuS-3 with excessive CuS loading, the CuS diffraction peaks become dominant. These results demonstrate that an appropriate CuS loading is crucial for achieving well-balanced MXene@CuS heterostructure with optimal structural properties.

FT-IR spectroscopy was conducted on MXene@CuS with varying CuS loadings to elucidate the surface chemical states and interfacial interactions (Fig. 1i). For all samples, characteristic absorption bands are observed around 3427 cm^−1^, corresponding to the O–H stretching vibrations of surface-adsorbed water and hydroxyl groups [27]. The absorption features at 1630 and 577 cm^−1^ correspond to the C–O stretching vibrations and Ti–O bond vibrations of MXene, respectively [28, 29]. Furthermore, distinct absorption bands at 2925 and 2850 cm^−1^ are assigned to the asymmetric and symmetric stretching vibrations of C–H bonds, respectively [30]. Additionally, Raman spectra of MXene@CuS samples exhibit distinct peaks near 154 cm^−1^ (E_g_), 392 cm^−1^ (B_1g_), and 625 cm^−1^ (E_g_), which correspond to the vibrational modes of anatase TiO_2_, indicating partial surface oxidation of MXene substrate (Fig. 1j) [31–33]. With increasing CuS loading, the intensities of MXene related peaks progressively decrease. Notably, the absence of extra Raman features in all samples further verifies the phase purity of the materials.

XPS was performed to characterize the surface chemical states and interfacial interactions within the optimized MXene@CuS-2 heterostructure (Fig. 1k). The C 1*s* spectrum can be deconvoluted into C–C (284.8 eV), C–O (286.8 eV), C=O (288.6 eV), and C–Ti (281.0 eV) peaks (Fig. S9) [34, 35]. Three doublets are identified in the high-resolution Ti 2*p* spectrum, with Ti 2*p*_1/2_ peaks located at 460.1, 461.5, and 464.3 eV and corresponding Ti 2*p*_3/2_ peaks at 454.5, 456.2, and 458.5 eV, which are assigned to Ti–C, Ti^2+^, and Ti–O species, respectively. The presence of characteristic Ti–O signals indicates partial surface oxidation, likely induced during the hydrothermal process, which is consistent with the XRD results [20, 21, 36, 37]. The O 1* s* spectrum further supports this interpretation, displaying peaks at 529.8 eV (Ti–O) and 532.7 eV (C = O), consistent with a partially oxidized MXene (Fig. S9). The high-resolution Cu 2*p* spectrum exhibits two characteristic peaks located at approximately 931.5 and 951.6 eV, corresponding to Cu 2*p*_3/2_ and Cu 2*p*_1/2_ peaks, respectively. Peak fitting confirms the coexistence of Cu^+^ and Cu^2+^, accompanied by characteristic satellite peaks [38–40]. The S 2*p* spectrum exhibits two main peaks at 160.5 eV (2*p*_3/2_) and 161.6 eV (2*p*_1/2_), while additional features may originate from surface-related or intermediate sulfide species formed during the reaction [38, 41, 42]. Additionally, TGA was conducted to evaluate the thermal stability of MXene@CuS (Fig. S10). The sample undergoes a weight loss of approximately 4.43% from room temperature to 800 °C, which is attributed to the removal of adsorbed water and oxidation of Ti–C framework in MXene, exhibiting good thermal stability.

### Thermophysical Property Analysis

**Fig. 2:**
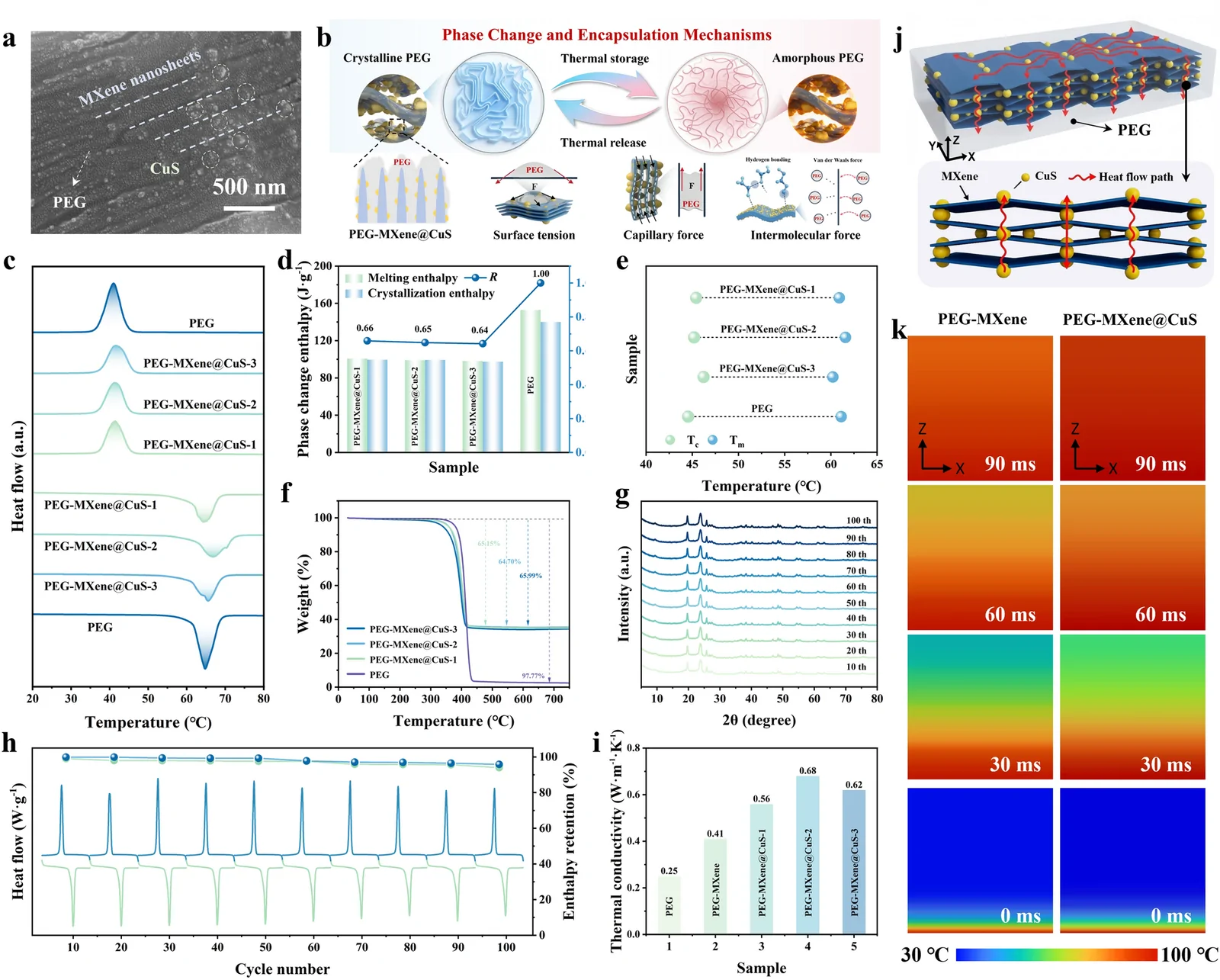
**a** SEM image of PEG-MXene@CuS-2. **b** Schematic illustration of phase change behavior and encapsulation mechanism of composite PCMs. **c** DSC curves showing the melting and crystallization processes of PEG and PEG-MXene@CuS. **d** Phase change enthalpy and encapsulation ratio. **e** Phase change temperatures. **f** TGA curves of PEG and PEG-MXene@CuS. **g** Cyclic XRD patterns. **h** Cyclic DSC curves and enthalpy retention of PEG-MXene@CuS-2. **i** Thermal conductivity of PEG, PEG-MXene and PEG-MXene@CuS. **j** Schematic heat transfer diagram of PEG-MXene@CuS. **k** Steady-state temperature fields of PEG-MXene and PEG-MXene@CuS using ANSYS fluent

To evaluate the encapsulation efficacy of PEG within the MXene@CuS, a typical leakage test was conducted (Fig. S11). PEG-MXene@CuS composite PCMs and pure PEG were placed on filter paper and heated to 80 °C (above the melting point of PEG) for 2 h. While pure PEG shows obvious liquid spreading and significant mass loss, PEG-MXene@CuS composite PCMs retain their original shape with no visible leakage, demonstrating the stable physical confinement of PEG via MXene@CuS. The original accordion-like and bridged morphology of MXene@CuS is well preserved (Figs. 2a and S12). PEG molecules infiltrate the interlayer gaps and coat the MXene@CuS surfaces, forming a smooth and continuous layer. The XRD patterns of PEG-MXene@CuS correspond to a superposition of the characteristic peaks from MXene@CuS and PEG (Fig. S13). No significant peak shifts or new diffraction peaks are detected, indicating that PEG is physically encapsulated into the interlayer spaces and pores of MXene@CuS without disrupting its crystal structure or forming new crystalline phases. FT-IR analysis further supports the aforementioned physical encapsulation mechanism (Fig. S14). The FT-IR spectrum of PEG-MXene@CuS exhibits a direct superposition of the characteristic absorption bands from MXene@CuS and PEG. No significant shifts or additional peaks are observed in any characteristic absorption peaks, indicating no strong chemical bonding between PEG and MXene@CuS. Their interaction is primarily governed by physical adsorption via hydrogen bonding and capillary effects.

The crystallization and melting behavior of PEG-MXene@CuS composite PCMs are synergistically regulated through the porous heterogeneous network and the multiple interactions with the PEG molecules (Fig. 2b). During the endothermic melting process, solid PEG absorbs heat and liquefies, with capillary forces effectively confining liquid PEG within the porous network formed by MXene and CuS to prevent leakage. Simultaneously, dynamic hydrogen bonding between the ether oxygen atoms in PEG chains and the oxygen-containing functional groups on MXene surfaces enhances interfacial adhesion, ensuring robust attachment of PEG to the porous heterogeneous network. In the subsequent exothermic crystallization stage, liquid PEG releases latent heat and gradually solidifies, during which surface tension maintains intimate contact with the porous network, promoting the formation of a uniform crystallization interface. Meanwhile, van der Waals forces between PEG chains and the surfaces of MXene nanosheets and CuS nanoparticles further provide extensive nonspecific adsorption. These forces act cooperatively with hydrogen bonds to guide the orderly arrangement and nucleation of PEG molecules, promoting homogeneous crystallization throughout the composite. The synergy of these interactions not only effectively suppresses PEG leakage and agglomeration but also optimizes crystallization behavior, ensuring a stable phase change enthalpy of PEG-MXene@CuS over long-term cycling.

Figure 2c presents the DSC curves of pure PEG and PEG-MXene@CuS composite PCMs. All PEG-MXene@CuS composites exhibit well-defined melting and crystallization peaks similar to those of pure PEG, with phase change temperatures showing minimal variation across different CuS loadings (Fig. 2e, Table S2). The phase change enthalpy of PEG-MXene@CuS composites is about 98–100 J g^−1^ (Fig. 2d). These results confirm that the introduction of CuS does not significantly alter the intrinsic phase change behavior of PEG, and the stable heterostructured network constructed by MXene and CuS effectively supports and preserves phase change capability of PEG. Furthermore, TGA curves (Fig. 2f) demonstrate superior thermal stability of PEG-MXene@CuS below 300 °C. To evaluate the long-term service reliability of PEG-MXene@CuS-2, we performed 100 heating–cooling cycles (Fig. 2h). The phase change enthalpy and temperature of PEG-MXene@CuS-2 remain almost unchanged, demonstrating excellent cyclic energy-storage stability (Fig. S15). XRD patterns of PEG-MXene@CuS-2 before and after cycling show no significant changes in the characteristic diffraction peaks of PEG, MXene, or CuS, indicating that the crystal structures of all components remain stable without phase separation or chemical degradation during repeated phase change (Fig. 2g). This conclusion is further supported by FT-IR spectra, which show no peak shift or new absorption bands after cycling (Fig. S16). Together, these results indicate that PEG-MXene@CuS-2 possesses good chemical stability, structural integrity, and thermal storage durability under long-term phase change cycling.

High thermal conductivity is crucial for achieving rapid thermal response and uniform temperature distribution during thermal storage and release of composite PCMs. As shown in Fig. 2i and Table S3, pure PEG exhibits a low thermal conductivity of only 0.25 W m^−1^ K^−1^. With the introduction of MXene forming a preliminary conductive network, PEG-MXene@CuS-1 shows an increased thermal conductivity of 0.56 W m^−1^ K^−1^. Upon further incorporation of CuS, PEG-MXene@CuS-2 demonstrates a significantly enhanced thermal conductivity of 0.68 W m^−1^ K^−1^. However, PEG-MXene@CuS-3 shows a reduced thermal conductivity of 0.62 W m^−1^ K^−1^ due to excessive CuS aggregation. This behavior can be explained by a CuS-bridged thermal conduction model. At an appropriate loading, CuS nanoparticles effectively attach to the surfaces and edges of MXene nanosheets, acting as thermal bridges between adjacent MXene layers (Fig. 2j). This structure not only fills the gaps between MXene sheets, reducing interfacial thermal resistance, but also constructs a more continuous 3D thermal transfer pathway, thereby significantly enhancing overall thermal conductivity. Insufficient CuS loading results in a limited number of thermal bridges and poor network connectivity, while excessive loading tends to cause agglomeration of CuS nanoparticles, which disrupts efficient heat flow. To visually confirm thermal transfer mechanism, the temperature fields of PEG-MXene and PEG-MXene@CuS-2 under identical boundary conditions were simulated using ANSYS Fluent (Fig. 2k). The results show that under heating, PEG-MXene@CuS-2 exhibits a more uniform temperature distribution, faster heat diffusion, and broader isotherm coverage, directly confirming the significant enhancement of overall heat transfer capability enabled by CuS as thermal bridges.

### Photothermal Conversion and Mechanism Analysis

**Fig. 3:**
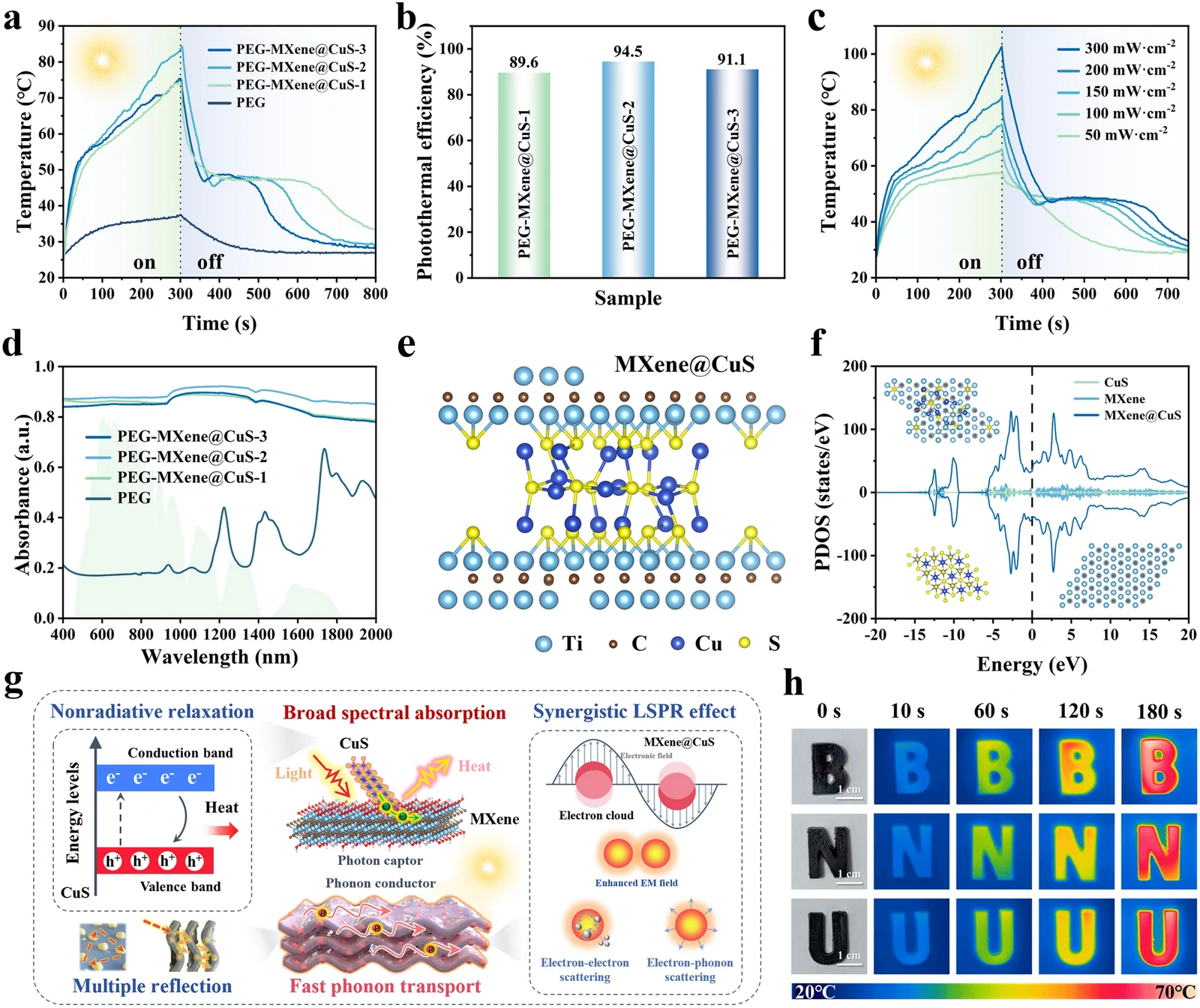
**a** Photothermal conversion curves and **b** corresponding conversion efficiencies of PEG-MXene@CuS (100 mW cm^−2^). **c** Photothermal conversion curves of PEG-MXene@CuS-2 under different irradiation intensities. **d** UV–Vis-NIR absorption spectra of PEG and PEG-MXene@CuS. **e** Optimized geometrical structures and **f** PDOS of MXene@CuS heterojunction. **g** Schematic illustration of photothermal conversion mechanism of PEG-MXene@CuS. **h** Infrared thermal images of PEG-MXene@CuS-2 during photothermal conversion under irradiation

To overcome the inherent intermittency of solar energy, it is particularly important to evaluate and optimize the photothermal conversion of PCMs. In this context, PEG‑MXene@CuS composites offer a promising pathway toward efficient, stable, and controllable solar energy utilization. Accordingly, photothermal conversion tests were conducted under simulated solar irradiation (100 mW cm^−2^), with temperature‑time profiles recorded to assess the thermal response. Compared with pure PEG, which shows only a gradual and limited temperature increase due to its poor light absorption, all PEG‑MXene@CuS composite PCMs exhibit significantly enhanced photothermal heating (Fig. 3a). Upon irradiation, PEG-MXene@CuS rapidly heats up, reflecting a swift photothermal response. The temperature then enters a relatively stable plateau region, corresponding to the continuous absorption of latent heat during the solid‑liquid phase change of PEG. This behavior confirms the effective conversion of photothermal energy into latent heat storage. Following the plateau, the temperature rises steadily again, indicating the accumulation of sensible heat after phase change completion. Upon switching off the light source, the temperature of PEG-MXene@CuS drops promptly due to efficient heat dissipation to the surroundings. The cooling curve subsequently transitions to a gently declining plateau, associated with the gradual release of latent heat during the liquid‑solid phase change of PEG. Comparatively, PEG-MXene@CuS-1 and PEG-MXene@CuS-3 exhibit a weaker photothermal response owing to sparse CuS distribution in PEG-MXene@CuS-1 and severe CuS agglomeration in PEG-MXene@CuS-3. Benefiting from its optimized thermal bridge heterostructure, PEG-MXene@CuS-2 exhibits the highest photothermal conversion efficiency of 94.5% (100 mW cm^−2^) (Fig. 3b, Table S4).

To evaluate optical intensity adaptability, the photothermal behavior of PEG-MXene@CuS-2 was systematically investigated over a light intensity range of 50–300 mW cm^−2^ (Fig. 3c). Both the heating profiles and the final equilibrium temperatures exhibit a clear dependence on incident light intensity, indicating its potential applicability in photothermal systems under varying irradiation conditions. Furthermore, PEG-MXene@CuS-2 underwent 10 consecutive heating–cooling cycles under periodic light on/off switching (Fig. S17). The sample exhibited excellent stability, with both the temperature rise and phase change plateau duration remaining consistent throughout all cycles. These results collectively demonstrate the superior photothermal stability and structural reliability of PEG-MXene@CuS-2 under practical operating conditions. Overall, PEG-MXene@CuS exhibited excellent photothermal conversion and thermoelectric generation capabilities compared with previously reported composite PCMs (Table S5).

To elucidate optical capture characteristics of PEG-MXene@CuS composite PCMs and their correlation with photothermal behavior, ultraviolet–visible-near-infrared (UV–Vis-NIR) absorption spectra were measured (Fig. 3d). Pure PEG exhibits negligible absorption across the 400–2000 nm range, confirming its inherently weak optical capture ability. In contrast, PEG-MXene@CuS demonstrates significantly enhanced broadband absorption, primarily attributed to the synergistic light-harvesting effect between MXene and CuS. To gain deeper insight into the underlying mechanism, first-principles density functional theory (DFT) calculations were performed. The results reveal the formation of a stable heterojunction at the MXene/CuS interface, accompanied by significant structure reorganization and charge transfer (Fig. 3e). PDOS further demonstrates that interfacial interactions between MXene and CuS significantly modulate the electronic states near the Fermi level (E_F_) (Fig. 3f). Given that light absorption originates from electron transitions and its efficiency is closely related to the availability of electronic states, low-energy photons can more readily induce electron transitions, thereby enhancing the optical response [43, 44]. As the metallic nature of Ti_3_C_2_T_x_, interfacial coupling with CuS facilitates more efficient electron excitation from the valence states of CuS to the hybridized states at the interface, thereby broadening the solar absorption spectrum.

Based on these findings, the photothermal conversion mechanism of PEG-MXene@CuS composite PCMs is fundamentally driven by synergistic localized surface plasmon resonance (LSPR) effects and tight interfacial coupling (Fig. 3g). Specifically, CuS nanoparticles, as a p-type semiconductor, exhibit intense LSPR in the near-infrared (NIR) region due to their high concentration of free holes [45]. In the composite heterostructure, the highly conductive 2D layered MXene functions as an efficient photon captor, enhancing the local electromagnetic field at the heterointerface and thereby amplifying the light absorption of CuS nanoparticles through a synergistic LSPR effect [46]. The strong interfacial coupling also promotes efficient charge excitation and energy transfer under light irradiation. Subsequently, the absorbed photon energy is converted into lattice vibrations through non-radiative relaxation, where excited carriers undergo rapid relaxation via enhanced electron–electron and electron–phonon scattering at the tightly coupled interface. Meanwhile, the layered MXene promotes multiple internal reflections to extend the optical path length, while the CuS-bridged 3D interconnected heterostructure acts as a phonon transporter, leveraging the high thermal conductivity of MXene to facilitate rapid thermal diffusion. Consequently, the combined effects of broadband absorption, LSPR enhancement, multiple reflections, and rapid phonon transport enable efficient solar energy conversion and stable latent heat storage. This is visually supported by infrared thermal imaging, which demonstrates uniform surface temperature distribution for PEG-MXene@CuS-2 under simulated solar irradiation (Fig. 3h).

### Photoresponsive Thermoelectric Cogeneration

**Fig. 4:**
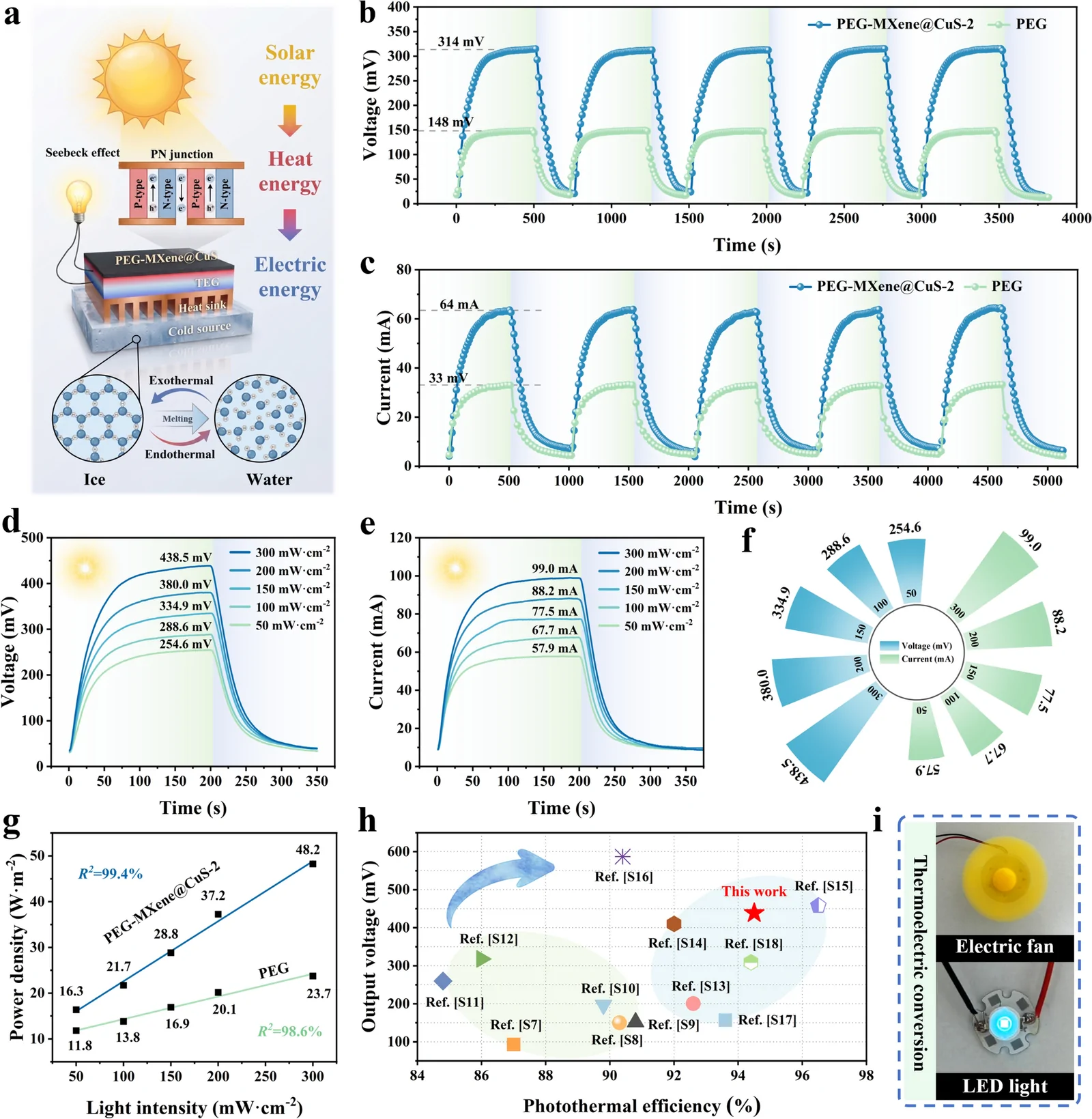
**a** Schematic diagram of the photothermoelectric conversion mechanism. **b** Output voltage and **c** output current of PEG and PEG-MXene@CuS-2 over 5 consecutive cycles. **d** Output voltage and **e** output current curve of PEG-MXene@CuS-2 across 50 to 300 mW cm^−2^. **f** Output voltage and current of PEG-MXene@CuS-2 under varying irradiation intensities. **g** Power densities of PEG and PEG-MXene@CuS-2 varying irradiation intensities. **h** Comparison of photothermal conversion efficiency and output voltage with reported composite PCMs. **i** Digital photograph demonstrating practical application

Building upon the efficient photothermal conversion, we further evaluated the photothermoelectric conversion capability to explore its potential for comprehensive solar energy utilization. Photothermoelectric conversion technology directly transforms the heat generated via PCMs into electricity. In this configuration, the illuminated PEG-MXene@CuS-2 serves as the hot side, while a constant temperature cold side is maintained using an ice water bath, with both sides connected through the commercial thermoelectric generator (TEG). Under simulated solar irradiation, PEG-MXene@CuS-2 can rapidly convert absorbed light into heat, raising the temperature at the hot side and thereby establishing a stable temperature difference across the TEG. Driven by the Seebeck effect, the temperature gradient promotes directional carrier migration within the thermoelectric device, generating a continuous voltage/current signal that was recorded in real time via electrochemical workstation (Fig. 4a). Mechanistically, the synergistic interaction between MXene and CuS endows composite PCMs with high photothermal conversion efficiency, enabling rapid heating of the hot side under illumination. During photothermoelectric conversion process, phase change behavior of PEG plays a critical role in dynamic thermal regulation. Specifically, the solid liquid phase change of PEG absorbs substantial latent heat, buffering excessive temperature rise at the hot side and helping to prolong the steady-state thermal gradient. Consequently, PEG-MXene@CuS-2 not only achieves efficient photothermal conversion but also, through its intrinsic thermal storage capability, optimizes the temperature stability and output continuity of the thermoelectric process.

To explore the photothermoelectric conversion, PEG-MXene@CuS-2 composite PCMs and pure PEG were subjected to five consecutive on off illumination cycles under 100 mW cm^−2^ (Fig. 4b, c). Upon illumination, the voltage and current of PEG-MXene@CuS-2 rapidly increase, reaching peak values of approximately 314 mV and 64 mA within a short period, attributed to its efficient photothermal conversion capability and the pronounced temperature difference across the thermoelectric device. In contrast, pure PEG exhibits weaker voltage and current responses, with significantly lower peak voltages/currents and slower rise rates, due to its limited photothermal conversion capacity. During sustained illumination, the output signals of composite PCMs remain at a stable plateau, while those of pure PEG stays at a low level throughout. Notably, over five consecutive cycles, the output amplitude and waveform of PEG-MXene@CuS-2 remain highly consistent without noticeable degradation, indicating excellent cyclic stability and reliability.

To evaluate the adaptability and output tunability of PEG-MXene@CuS-2 under realistic fluctuating solar irradiation, its voltage time and current time profiles were systematically measured at different light intensities (50–300 mW cm^−2^) (Fig. 4d, e). The results show that as the light intensity increases from 50 to 300 mW cm^−2^, the steady-state output voltage rises from 254.6 to 438.5 mV, while the steady-state output current increases from 57.9 to 99.0 mA (Table S6). Notably, the output voltage and current exhibit a clear positive correlation with light intensity, and the response is accelerated with higher irradiance. Statistical analysis of the steady-state outputs under different intensities further confirms a strong linear relationship (*R*^*2*^ > 0.99) between both voltage/current and irradiation intensity (Figs. 4f and S18). Based on the linear fitting, the output power density of PEG-MXene@CuS-2 is plotted as a function of light intensity (Fig. 4g), reaching 48.2 W m^−2^ at 300 mW cm^−2^. PEG-MXene@CuS-2 composite PCMs deliver stable and tunable electrical output across a wide range of light intensities, which is primarily attributed to the synergistic interplay between the photothermal conversion of MXene@CuS heterojunction for rapidly establishing a temperature gradient and thermal storage behavior of PEG for effectively buffering thermal fluctuations. Thus, PEG-MXene@CuS-2 composite PCMs not only can adapt to high intensity irradiation for high-power output, but also operates stably under low light conditions, providing a reliable foundation for practical use in fluctuating solar environments. Additionally, Fig. 4h compares the photothermal conversion efficiency and output voltage of PEG-MXene@CuS-2 with recently reported photoresponsive composite PCMs. To visually demonstrate photothermoelectric capability, PEG-MXene@CuS-2 is further integrated into a simple circuit. Under simulated sunlight, it successfully powers a commercial LED and a small DC fan, directly confirming its ability to effectively convert light into sufficient electrical energy to drive microelectronic devices (Fig. 4i).

### Microwave Absorption and Mechanism Analysis

**Fig. 5:**
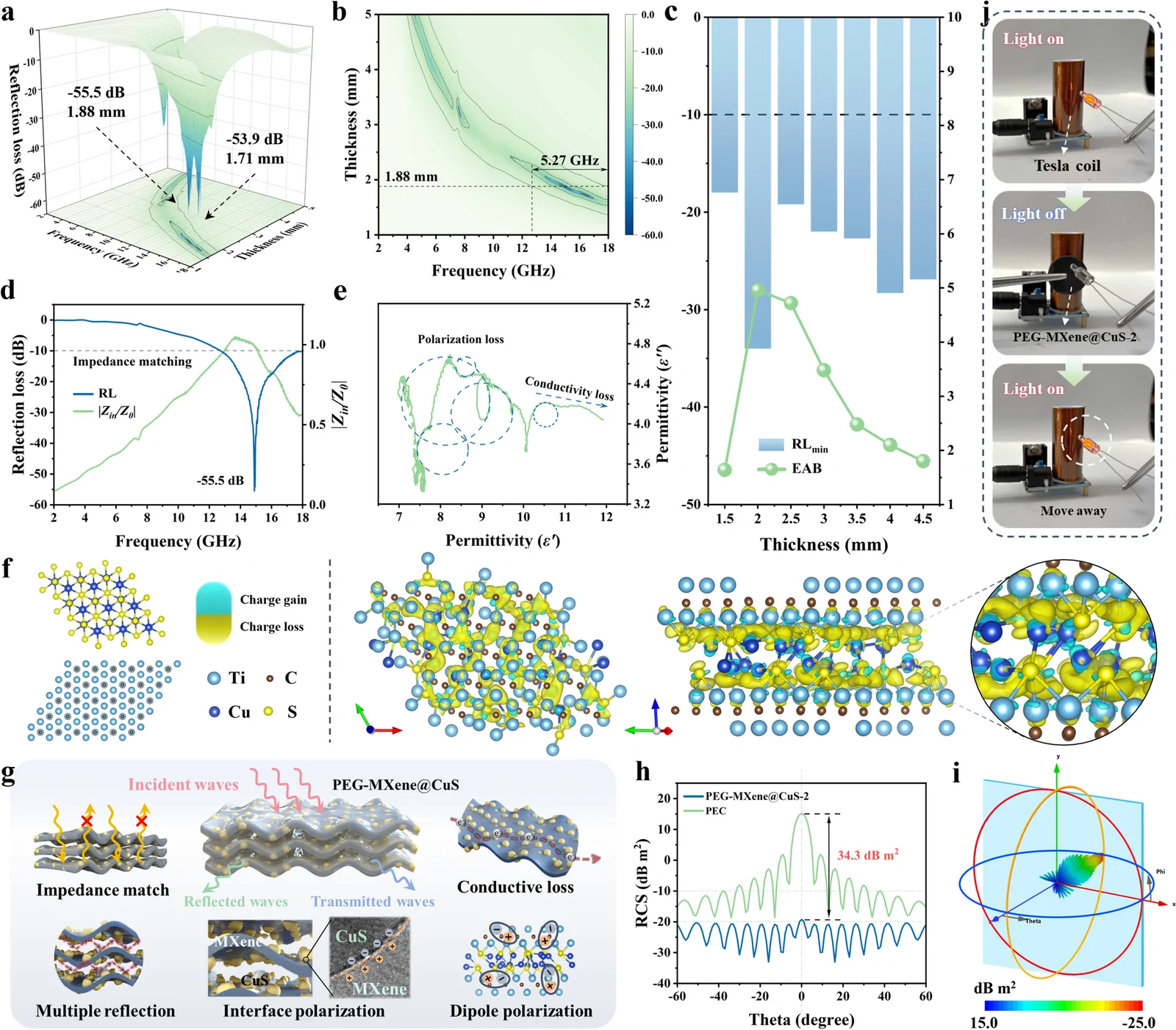
**a** 3D representation and **b** 2D projection of the RL for PEG-MXene@CuS-2. **c** RL_min_ and EAB statistics of PEG-MXene@CuS-2 with different thicknesses. **d** Relationship between RL_min_ and |*Z*_in_*/Z*_0_| of PEG-MXene@CuS-2 at 1.88 mm. **e** Cole–Cole plot of PEG-MXene@CuS-2. **f** Differential charge density diagram of MXene@CuS. **g** Schematic of microwave absorption mechanisms for PEG-MXene@CuS. **h** RCS simulation results of samples at different angles. **i** 3D diagrams of RCS simulations for PEC coated with PEG-MXene@CuS-2. **j** Microwave absorption test using a Tesla coil-LED device

Developing high-performance microwave absorption PCMs is crucial for applications in electromagnetic protection, military stealth, and next-generation communications. To systematically evaluate the microwave absorption of PEG-MXene@CuS-2, we measured key electromagnetic parameters, including the complex permittivity (*ε′*, *ε″*) and complex permeability (*μ′*, *μ″*) (Fig. S19). Notably, in contrast to the significant values of complex permittivity, the *μ′* and *μ″* values are close to 1.0 and 0, respectively, indicating that the composite is approximately non-magnetic and its microwave attenuation is primarily governed by dielectric loss [47]. Further, we calculated reflection loss (RL) and effective absorption bandwidth (EAB), which confirm that PEG-MXene@CuS-2 exhibits optimal performance due to its moderate CuS loading (Fig. S20). The lower RL indicates stronger microwave absorption, while EAB is defined as the frequency range over which RL ≤ -10 dB, corresponding to > 90% absorption of incident microwaves. Figure 5a, b shows 3D and 2D RL contour plots of PEG-MXene@CuS-2, achieving an RL_min_ of -55.5 dB and an EAB as high as 5.27 GHz at a thickness of 1.88 mm (Fig. 5c). These results demonstrate its competitive performance compared with previously reported composite PCMs (Table S7).

To elucidate the underlying physical mechanisms, we systematically analyzed electromagnetic loss characteristics, impedance matching behavior, and interfacial interactions. The dielectric loss tangent (tan*δ*_*ε*_ = *ε″/ε′*) and magnetic loss tangent (tan*δ*_*μ*_ = *μ″/μ′*) were calculated from the complex permittivity and permeability (Fig. S21). Results indicate that dielectric loss dominates across the entire 2–18 GHz range, which is attributed to abundant polarization relaxation centers introduced by MXene/CuS heterointerface and the intrinsic conductive loss of MXene. Furthermore, modulation of the dielectric constant effectively regulates the impedance matching of the PEG-MXene@CuS composites. Generally, a normalized input impedance (*|Z*_in_*/Z*_0_*|*) approaching 1 indicates that incident waves can penetrate the absorber with minimal surface reflection [18, 48]. The optimized CuS interfacial bridging expands the impedance matching region (0.8 ≤*|Z*_in_*/Z*_0_*|*≤ 1.2), suggesting that this heterostructure engineering optimizes impedance matching, thereby enabling more electromagnetic waves to penetrate the material and facilitating further energy dissipation (Fig. S22). In addition, the correlation between |Z_in_/Z_0_| and RL values provides a more direct depiction of their relationship, confirming that proper impedance matching is a prerequisite for achieving superior microwave absorption (Fig. 5d).

To gain deeper insights into the dielectric loss mechanisms of PEG-MXene@CuS-2, the Cole–Cole plot was systematically analyzed based on the Debye relaxation theory. The curves consist of several distinct semicircular arcs and a linear tail, confirming the synergistic contribution of multiple relaxation processes and conductive loss (Fig. 5e) [49, 50]. By quantitatively fitting the relaxation parameters, three primary relaxation times were determined: *τ*_*1*_ = 1.96 × 10^–10^ s, *τ*_*2*_ = 2.60 × 10^–9^ s, and *τ*_*3*_ = 5.11 × 10^–11^ s, which correspond to interfacial polarization at MXene/CuS heterointerfaces and dipole polarization from surface functional groups (Fig. S23). Furthermore, by decomposing the imaginary part of the complex permittivity into conductive loss (*ε*_*c*_*''*) and polarization loss (*ε*_*p*_*''*), it is observed that polarization loss dominates across the entire frequency range, serving as the core mechanism for electromagnetic energy dissipation (Fig. S24). This characteristic, featuring dominant polarization loss complemented by conductive loss, originates from the abundant heterostructures formed by CuS bridging and the well-defined conductive network of MXene, which collectively facilitate the efficient attenuation of incident microwaves.

To further investigate the electronic structural evolution at the MXene/CuS interface, DFT calculations and differential charge density analysis were conducted (Fig. 5f). The results demonstrate significant charge redistribution at the MXene/CuS interface, characterized by spontaneous electron migration from MXene to CuS, as confirmed by the Cu 2*p* XPS spectrum, which indicates the formation of reduced Cu^+^ species. This directional transfer establishes a built-in electric field directed from MXene to CuS, which effectively modulates the interfacial electronic environment and facilitates the accumulation and polarization of charges under external electromagnetic excitation [51, 52]. Consequently, this electronic regulation promotes intense interfacial polarization and multi-polarization relaxation processes, which significantly enhance electromagnetic energy dissipation.

Therefore, the exceptional broadband microwave absorption of PEG-MXene@CuS-2 originates from a complex synergy of optimized impedance matching and enhanced dissipation mechanisms (Fig. 5g). First, the integration of MXene and CuS optimizes the complex electromagnetic parameters, yielding a normalized input impedance (*|Z*_in_*/Z*_0_*|*) close to 1 across a broad frequency range, which minimizes surface reflection and facilitates maximum wave penetration. Secondly, the CuS-bridged 3D interconnected network provides efficient pathways for free-carrier migration, contributing to substantial conductive loss. Crucially, as confirmed by DFT calculations, the spontaneous electron migration from MXene to CuS establishes a robust internal electric field at the heterointerface, which effectively modulates the local electronic environment, thereby promoting intense charge accumulation and interfacial polarization under external electromagnetic excitation. Consequently, polarization loss dominates the energy dissipation process, as evidenced by the multiple Debye relaxation cycles and the dominance of the polarization loss component (*ε*_*p*_*''*) over the conductive loss component (*ε*_*c*_*''*). Once inside the composite, microwaves also undergo multiple internal reflections and scattering within the hierarchical heterointerface network, significantly extending the propagation path and enhancing energy attenuation. In summary, through the synergistic effects of optimized impedance matching, enhanced interfacial polarization, and multiple scattering effects, PEG-MXene@CuS-2 achieves superior microwave absorption performance characterized by strong attenuation, broad bandwidth, and a thin matching thickness.

To visually demonstrate the practical application potential of PEG-MXene@CuS-2 composite PCMs, we conducted numerical simulations and experimental validation. Far field radar cross-section (RCS) simulations were conducted using CST Studio Suite 2024 for a model structure coated with PEG-MXene@CuS-2. Compared with bare perfect electric conductor (PEC) substrate, the coated structure significantly reduces the scattering of 3D radar waves. Under normal incidence (0°), the RCS of PEG-MXene@CuS-2 is reduced by 34.3 dB m^2^ relative to the PEC substrate, confirming its effectiveness for microwave absorption applications (Fig. 5h, i). Additionally, a straightforward Tesla coil demonstration was designed to visually verify the microwave absorption capability. A light emitting diode (LED), sensitive to high frequency electromagnetic fields, was placed within the strong radiation field generated via the Tesla coil. When approaching, the LED lights up continuously, whereas it dims rapidly once covered with a thin layer of PEG-MXene@CuS-2, demonstrating its excellent electromagnetic wave management capability (Fig. 5j). Furthermore, the samples retain nearly identical electromagnetic parameters after the phase transition cycle, demonstrating the reliability of the material for practical applications (Fig. S25).

## Conclusions

In this work, advanced multifunctional PEG-MXene@CuS composite PCMs were constructed through a synergistic strategy combining interface bridging engineering and physical encapsulation. By in-situ growing CuS nanoparticles as interlayer pillars on 2D MXene, a 3D interpenetrating phonon/electron transport network was established, which effectively alleviates the intrinsic impedance mismatch and high interfacial thermal resistance prevalent in MXene-based hybrids while providing stable physical confinement for PEG. The resulting PEG-MXene@CuS composite PCMs exhibit exceptional comprehensive performance, achieving a high photothermal conversion efficiency of 94.5% under 100 mW cm^−2^ irradiation and delivering a stable power output of 21.7 W m^−2^ when integrated with a thermoelectric module. Moreover, benefiting from optimized impedance matching and enhanced interfacial polarization, PEG-MXene@CuS demonstrates remarkable microwave absorption, with a minimum reflection loss of -55.5 dB and an effective absorption bandwidth of 5.27 GHz at a thickness of only 1.88 mm. Notably, the phase change enthalpy retention exceeds 95% after multiple melting-crystallization cycles, highlighting its excellent cyclic stability and long-term reliability. This study provides important insights into developing multifunctional integrated materials that combine efficient photothermal conversion, stable thermal storage, reliable thermoelectric output, and broadband microwave absorption, showing promising potential for applications in intelligent thermal management, solar energy utilization, and electromagnetic compatibility.

## Supplementary Information

Below is the link to the electronic supplementary material.

Supplementary file1 (DOCX 14740 kb)

## References

[1] H. Lv, Z. Yang, H. Pan, R. Wu, Electromagnetic absorption materials: current progress and new frontiers. Prog. Mater. Sci. **127**, 100946 (2022). 10.1016/j.pmatsci.2022.100946

[2] A. Pandey, S. Panda, S. Mehlawat, N. Dhariwal, A. Sanger, Breaking detection barriers: next-generation dual-band radar/IR stealth materials- breakthroughs, challenges, and the revolution in advanced defense platforms. Coord. Chem. Rev. **557**, 217731 (2026). 10.1016/j.ccr.2026.217731

[3] Z. Zhao, Y. Qing, L. Kong, H. Xu, X. Fan et al., Advancements in microwave absorption motivated by interdisciplinary research. Adv. Mater. **36**(4), 2304182 (2024). 10.1002/adma.202304182 10.1002/adma.202304182 37870274

[4] Z. Nan, W. Wei, Z. Lin, J. Ouyang, J. Chang et al., Flexible electromagnetic interference shields: materials, structure and multifunctionalization. Mater. Sci. Eng. R. Rep. **160**, 100823 (2024). 10.1016/j.mser.2024.100823

[5] M.M. Rahman, S. Imani, N. Anjum, A.A. Sijuade, O. Okoli, Materials and design strategies for next-generation energy storage: a review. Renew. Sustain. Energy Rev. **212**, 115368 (2025). 10.1016/j.rser.2025.115368

[6] H. Li, L. Yan, J. Zhou, Y. Wang, X. Liao et al., Flexible and wearable functional materials for ionizing radiation protection: a perspective review. Chem. Eng. J. **487**, 150583 (2024). 10.1016/j.cej.2024.150583

[7] Y. Li, M. Zhang, J. Chen, X. Liu, M. Huang et al., Ceramic-based electromagnetic interference shielding materials: mechanisms, optimization strategies, and pathways to next-generation applications. J. Adv. Ceram. **14**(12), 9221194 (2025). 10.26599/jac.2025.9221194

[8] S. Chen, S. Fan, Z. Qiao, Z. Wu, B. Lin et al., Transforming healthcare: intelligent wearable sensors empowered by smart materials and artificial intelligence. Adv. Mater. **37**(21), 2500412 (2025). 10.1002/adma.202500412 40167502 10.1002/adma.202500412 PMC12107229

[9] Z. Sun, S. Tian, Z. Guo, J. Bi, J. Wang et al., Multi-stage anisotropic cellulose-based aerogels *via* “foam-flake-fiber” interweaving for versatile microwave absorbers. Adv. Funct. Mater. **35**(44), 2508255 (2025). 10.1002/adfm.202508255

[10] S. Zheng, W. Xu, J. Liu, F. Pan, S. Zhao et al., One-hour ambient-pressure-dried, scalable, stretchable MXene/polyurea aerogel enables synergistic defense against high-frequency mechanical shock and electromagnetic waves. Adv. Funct. Mater. **34**(38), 2402889 (2024). 10.1002/adfm.202402889

[11] J.-Y. Zong, Z.-Z. Wang, W.-Q. Cao, M.-S. Cao, Flexible and lightweight hollow MXene@Carbon microfiber composites for microwave absorption and light-responsive devices. Adv. Funct. Mater. **36**(39), e74876 (2026). 10.1002/adfm.74876

[12] Q. Liang, M. He, B. Zhan, H. Guo, X. Qi et al., Yolk-shell CoNi@N-doped carbon-CoNi@CNTs for enhanced microwave absorption, photothermal, anti-corrosion, and antimicrobial properties. Nano-Micro Lett. **17**(1), 167 (2025). 10.1007/s40820-024-01626-8 10.1007/s40820-024-01626-8 PMC11865380 40009269

[13] I. Hussain, F. Bibi, S. Pandiyarajan, A. Hanan, H.-C. Chuang et al., Partially oxidized MXenes for energy storage applications. Prog. Mater. Sci. **147**, 101351 (2025). 10.1016/j.pmatsci.2024.101351

[14] R. Kumar, S. Sahoo, E. Joanni, J.-J. Shim, Cutting edge composite materials based on MXenes: synthesis and electromagnetic interference shielding applications. Compos. B Eng. **264**, 110874 (2023). 10.1016/j.compositesb.2023.110874

[15] C. Guo, S. Shao, X. Zhang, Y. Tang, L. Wang et al., Multifunctional MXene/rGO aerogels loaded with Co/MnO nanocomposites for enhanced electromagnetic wave absorption, thermal insulation and pressure sensing. Nano Res. **17**(9), 7803–7813 (2024). 10.1007/s12274-024-6840-x

[16] P. Huang, W.-Q. Han, Recent advances and perspectives of lewis acidic etching route: an emerging preparation strategy for MXenes. Nano-Micro Lett. **15**(1), 68 (2023). 10.1007/s40820-023-01039-z 10.1007/s40820-023-01039-z PMC10014646 36918453

[17] D. Xu, Z. Li, L. Li, J. Wang, Insights into the photothermal conversion of 2D MXene nanomaterials: synthesis, mechanism, and applications. Adv. Funct. Mater. **30**(47), 2000712 (2020). 10.1002/adfm.202000712

[18] Z. Tian, F. Hu, P. Zhang, Y. Fan, A.S. Shamshirgar et al., High-entropy engineering of A-site in MAX phases toward superior microwave absorption properties. Matter **8**(12), 102367 (2025). 10.1016/j.matt.2025.102367

[19] X. Wang, X. Chen, Q. He, Y. Hui, C. Xu et al., Bidirectional, multilayer MXene/polyimide aerogels for ultra-broadband microwave absorption. Adv. Mater. **36**(36), e2401733 (2024). 10.1002/adma.202401733 39039743 10.1002/adma.202401733

[20] F. Su, Z. He, J. Xie, J. Zhang, W. Zhang et al., Ti_3_C_2_T_*x*_ and copper sulfide composite nanofluids with a hierarchical structure for sustainable and efficient solar light–thermal conversion. J. Mater. Chem. A **11**(38), 20651–20664 (2023). 10.1039/D3TA03908K

[21] X. Ren, F. Meng, X. Meng, Ti_3_C_2_T_*x*_/CuS absorbent based on in situ growth strategy for wide frequency microwave absorption. Ceram. Int. **51**(27), 55011–55019 (2025). 10.1016/j.ceramint.2025.09.226

[22] G. Wang, Z. Tang, Y. Gao, P. Liu, Y. Li et al., Phase change thermal storage materials for interdisciplinary applications. Chem. Rev. **123**(11), 6953–7024 (2023). 10.1021/acs.chemrev.2c00572 36946191 10.1021/acs.chemrev.2c00572

[23] Y. Feng, K. Chen, P. Liu, J. Zhao, Y. Li et al., Longitudinal confinement engineering in phase change materials. eScience **6**(1), 100454 (2026). 10.1016/j.esci.2025.100454

[24] F. Zhao, W. Yuan, H. Chen, H. Fu, Z. Li et al., Advances in organic porous polymeric-supported photothermal phase change materials. Carbon Energy **7**(6), e719 (2025). 10.1002/cey2.719

[25] Y. Li, Y. Feng, M. Qin, K. Chen, Y. An et al., Co-anchored hollow carbonized kapok fiber encapsulated phase change materials for upgrading photothermal utilization. Small **21**(21), 2500479 (2025). 10.1002/smll.202500479 10.1002/smll.202500479 40166806

[26] Y. Li, X. Wang, K. Chen, Y. Feng, P. Liu et al., Hierarchical MoS_2_/CuS photonic nanostructure accelerating photothermoelectric conversion of bacterial cellulose based phase change materials. J. Colloid Interface Sci. **702**(Pt 1), 138863 (2026). 10.1016/j.jcis.2025.138863 40902488 10.1016/j.jcis.2025.138863

[27] T. Hassan, A. Iqbal, B. Yoo, J.Y. Jo, N. Cakmakci et al., Multifunctional MXene/carbon nanotube Janus film for electromagnetic shielding and infrared shielding/detection in harsh environments. Nano-Micro Lett. **16**(1), 216 (2024). 10.1007/s40820-024-01431-3 10.1007/s40820-024-01431-3 PMC11178741 38874857

[28] S. Zhang, F. Hu, P. Zeng, P. Hu, P. Zhang et al., Multiscale mechanically–electromagnetically coupled aerogels for tunable electromagnetic wave absorption. Compos. B Eng. **315**, 113518 (2026). 10.1016/j.compositesb.2026.113518

[29] Y. Xie, X. Xuan, Y. Tang, Z. Bi, P. Wang et al., Synergistic Mo/V-implanted 2D M3X2 MXene nanoarchitectures for enhanced structural stability and ultrahigh proton storage performance. Adv. Energy Mater. **16**(5), e05156 (2026). 10.1002/aenm.202505156

[30] J. Du, T. Li, J. Li, J. Tang, R. Zhang et al., Design of flexible MXene/graphene-based fiber fabrics for broadband electromagnetic wave absorption. Adv. Fiber Mater. **7**(3), 811–826 (2025). 10.1007/s42765-025-00523-y

[31] X. Zhu, X. Qian, M. Hao, Y. Zhang, Z. Zhang et al., High performance electromagnetic wave absorbing material based on 3D flower-liked MXene. J. Alloys Compd. **989**, 174440 (2024). 10.1016/j.jallcom.2024.174440

[32] Y. Liang, S. Cao, Q. Wei, R. Zeng, J. Zhao et al., Reversible Zn^2+^ insertion in tungsten ion-activated titanium dioxide nanocrystals for electrochromic windows. Nano-Micro Lett. **13**(1), 196 (2021). 10.1007/s40820-021-00719-y 10.1007/s40820-021-00719-y PMC8440694 34523029

[33] B. Lu, G. Jin, Y. Cui, T. Zhang, S. Liu et al., Carbon dots intercalated MXene for flexible organic hydrogel absorbers with synergistically enhanced dielectric loss. Nano-Micro Lett. **18**(1), 302 (2026). 10.1007/s40820-026-02135-6 10.1007/s40820-026-02135-6 PMC13018520 41880121

[34] T. Li, L. Ma, T. Chen, T. Yang, R. Qin et al., Interfacially engineered MXene-LDH 2D/2D heterostructures for integrated microwave absorption and corrosion protection. Nano Mater. Sci. (2026). 10.1016/j.nanoms.2026.02.010

[35] M. He, X. Lv, H. Peng, Y. Zhou, H. Li et al., Biomimetic artificial nacre-like microfiber of Co/C modified cellulose nanofiber/Ti_3_C_2_T_*x*_ MXene with efficient microwave absorption. Chem. Eng. J. **491**, 151726 (2024). 10.1016/j.cej.2024.151726

[36] Z. Cheng, Y. Xu, X. Zhang, Q. Peng, K. Wang et al., An interfacial covalent bonding coupled ultrafine CuS-nanocrystals/MXene heterostructure for efficient and durable magnesium storage. J. Mater. Chem. A **11**(23), 12176–12184 (2023). 10.1039/D3TA02416D

[37] Z. Wu, X. Tan, J. Wang, Y. Xing, P. Huang et al., MXene hollow spheres supported by a C-co exoskeleton grow MWCNTs for efficient microwave absorption. Nano-Micro Lett. **16**(1), 107 (2024). 10.1007/s40820-024-01326-3 10.1007/s40820-024-01326-3 PMC10837412 38305954

[38] M. Han, D. Lan, Z. Zhang, Y. Zhao, J. Zou et al., Micro-sized hexapod-like CuS/Cu_9_S_5_ hybrid with broadband electromagnetic wave absorption. J. Mater. Sci. Technol. **214**, 302–312 (2025). 10.1016/j.jmst.2024.07.014

[39] Y. Wu, Y. Zhao, M. Zhou, S. Tan, R. Peymanfar et al., Ultrabroad microwave absorption ability and infrared stealth property of nano-micro CuS@rGO lightweight aerogels. Nano-Micro Lett. **14**(1), 171 (2022). 10.1007/s40820-022-00906-5 10.1007/s40820-022-00906-5 PMC9392679 35987861

[40] J. Liu, L. Wu, J. Zhao, X. Liu, Y. Wu et al., Constructing multi-interfaced and vacancy-rich Cu_1.8_S/rGO/oleylamine composites toward anti-biofouling microwave absorption. Small **21**(17), 2412835 (2025). 10.1002/smll.202412835 10.1002/smll.202412835 40109166

[41] W. Wang, L. Cao, Q. Li, C. Du, S. Chen, Copper sulfide anchored MXene improving photo-responsive self-healing polyurethane with enhanced mechanical and antibacterial properties. J. Colloid Interface Sci. **630**(Pt B), 511–522 (2023). 10.1016/j.jcis.2022.10.089 36334487 10.1016/j.jcis.2022.10.089

[42] D. Gao, Y. Fu, B. Lyu, L. Tang, Z. Jia et al., Multifunctional wearable copper sulfide decorated leather nanocomposites towards efficient personal thermal management. Chem. Eng. J. **504**, 158918 (2025). 10.1016/j.cej.2024.158918

[43] C. Guo, X. Zhang, L. Wang, Y. Tang, H. Wang et al., Densely-neighbored-Ru nanoparticles confined in porous-SiO_2_ shell for efficient CO_2_ methanation *via* plasmon-coupling-enhanced photo-thermal catalysis. Sci. Bull. **70**(21), 3534–3543 (2025). 10.1016/j.scib.2025.09.038 10.1016/j.scib.2025.09.038 41047311

[44] Y. Tang, H. Wang, C. Guo, L. Wang, T. Zhao et al., Synergies between atomically dispersed Ru single atoms and nanoparticles on CeAlO_*x*_ for enhanced photo-thermal catalytic CO_2_ hydrogenation. Adv. Mater. **38**(1), e12793 (2026). 10.1002/adma.202512793 40937878 10.1002/adma.202512793

[45] Q. Bai, M. Liang, W. Wu, C. Zhang, X. Li et al., Plasmonic nanozyme of graphdiyne nanowalls wrapped hollow copper sulfide nanocubes for rapid bacteria-killing. Adv. Funct. Mater. **32**(20), 2112683 (2022). 10.1002/adfm.202112683

[46] T. Xu, S. Tan, S. Li, T. Chen, Y. Wu et al., Synergistic densification in hybrid organic-inorganic MXenes for optimized photothermal conversion. Adv. Funct. Mater. **34**(29), 2400424 (2024). 10.1002/adfm.202400424

[47] X. Meng, J. Qiao, J. Liu, L. Wu, Z. Wang et al., Bioinspired hollow/hollow architecture with flourishing dielectric properties for efficient electromagnetic energy reclamation device. Small **20**(11), 2307647 (2024). 10.1002/smll.202307647 10.1002/smll.202307647 37890470

[48] F. Hu, P. Ding, F. Wu, P. Zhang, W. Zheng et al., Novel cable-like tin@carbon whiskers derived from the Ti_2_SnC MAX phase for ultra-wideband electromagnetic wave absorption. Carbon Energy **6**(12), e638 (2024). 10.1002/cey2.638

[49] X. Meng, J. Qiao, J. Liu, L. Wu, Z. Wang et al., Core–shell nanofibers/polyurethane composites obtained through electrospinning for ultra-broadband electromagnetic wave absorption. Adv. Compos. Hybrid Mater. **7**(5), 149 (2024). 10.1007/s42114-024-00976-6

[50] X. Zhang, S. Xing, S. Shao, Y. Tang, Z. Wang et al., Rational micro/nano-architecture design of Fe_3_O_4_@TiO_2_/PI/rGO aerogels for integrated broadband microwave absorption, flame retardancy, and moisture resistance. Carbon **245**, 120840 (2025). 10.1016/j.carbon.2025.120840

[51] S. Zhang, J. Zheng, Z. Zhao, S. Du, D. Lan et al., New prospects in built-In electric fields for electromagnetic wave absorption: from fundamentals to interdisciplinary applications. Adv. Funct. Mater. **36**(1), e13762 (2026). 10.1002/adfm.202513762

[52] F. Hu, P. Zhang, P. Ding, S. Zhang, B. Fan et al., Magnetic-dielectric synergy in one-dimensional metal heterostructures for enhanced low-frequency microwave absorption. Nano-Micro Lett. **18**(1), 155 (2026). 10.1007/s40820-025-01995-8 10.1007/s40820-025-01995-8 PMC12765767 41486310

[53] X. Fan, L. Liu, X. Jin, W. Wang, S. Zhang et al., MXene Ti_3_C_2_T_*x*_ for phase change composite with superior photothermal storage capability. J. Mater. Chem. A **7**(23), 14319–14327 (2019). 10.1039/C9TA03962G

