# Supplementary material for: CuS-Bridged MXene-Based Photoresponsive Phase Change Materials Enabling Thermoelectric Cogeneration and Microwave Absorption
Source: Nanomicro Lett. 2026 Jul 7;18:428. doi: 10.1007/s40820-026-02277-7 (PMC13342011; doi:10.1007/s40820-026-02277-7)
Supplement: Supplementary file 1 — Supplementary file1 (DOCX 14740 kb) [file 40820_2026_2277_MOESM1_ESM.docx]

Supporting Information for

**CuS-bridged MXene-based** **Photoresponsive Phase Change Materials Enabling** **Thermoelectric Cogeneration and Microwave Absorption**

Yuhao Feng^1,^ ^†^, Jindi Zhao^1, †^, Keke Chen^1^, Yang Li^2,^ *, Jiao Liu^3^, Huitao Yu^4^, Mulin Qin^2^, Kaihang Jia^2^, Haiwei Han^2^, Zhenghui Shen^2^, Mingliang Ma^3,^ *, and Xiao Chen^1,^ *

^1^ School of Physics and Astronomy, Beijing Normal University, Beijing 100875, China

^2^ School of Materials Science and Engineering, Peking University, Beijing 100871, China

^3^ School of Civil Engineering, Qingdao University of Technology, Qingdao, 266033, China

^4^ School of Materials Science and Engineering, Inner Mongolia University of Science and Technology, Inner Mongolia 014010, China

^†^ These authors contributed equally to this work.

*Corresponding author. E-mail: younglee@pku.edu.cn, mamingliang@qut.edu.cn, xiaochen@bnu.edu.cn

**S1 Supplementary Calculation Formula**

**S1.1 Thermal Storage Calculation Formula**

The calculation formula of impregnation ratio (*R*), are as follows.

$$\begin{aligned} R=\frac{H_{m, PCMs}}{H_{m,PEG}} \#\left( 1 \right) \end{aligned}$$

where *H_m, PCMs_* represent the melting and freezing enthalpy of composite PCMs, respectively. Similarly, *H_m, PEG_* represent the melting and freezing enthalpy of PEG.

**S1.2 Photothermal Conversion Calculation Formula**

The photothermal conversion efficiency (*η*) was calculated according to the equation as follows:

$$\begin{aligned} \eta=\frac{m\Delta H_{m}}{PS\left( t_{2}-t_{1} \right)}\#\left( 2 \right) \end{aligned}$$

Where *m* and *S* denote mass and surface area of PEG-MXene@CuS composite PCMs, respectively; *P* represents the solar irradiance; and *t_1_* and *t_2_* signify the onset and termination times of the phase transition.

**S1.3 Microwave Absorption Calculation Formula**

***S1.3.1 Electromagnetic Parameters***

Electromagnetic parameters are crucial for evaluating the electromagnetic performance of electromagnetic wave absorbing materials. The dielectric and magnetic properties of materials can be characterized by the complex permittivity (*ε_r_*) and complex permeability (*μ_r_*):

$$\begin{aligned} \varepsilon_{r}=\varepsilon_{r}^{'}-j\varepsilon_{r}^{''}\#\left( 3 \right) \end{aligned}$$

$$\begin{aligned} \mu_{r}=\mu_{r}^{'}-j\mu_{r}^{''}\#\left( 4 \right) \end{aligned}$$

where *ε_r_'* denotes the real part of the complex permittivity, *ε_r_''* denotes the imaginary part of the complex permittivity, *μ_r_'* denotes the real part of the complex permeability, and *μ_r_''* denotes the imaginary part of the complex permeability. The real parts represent the ability to store electrical and magnetic energy, while the imaginary parts represent the ability to dissipate electrical and magnetic energy. For electromagnetic wave absorbing materials, larger values of the imaginary parts of *ε_r_* and *μ_r_* imply greater attenuation of incident electromagnetic wave, thereby indicating better electromagnetic wave absorption performance. Typically, the degree of electromagnetic wave loss is characterized by the dielectric loss tangent (*tanδ_ε_*) and magnetic loss tangent (*tanδ_μ_*):

$$\begin{aligned} \tan\delta_{\varepsilon}=\frac{\varepsilon^{''}}{\varepsilon^{'}}\#\left( 5 \right) \end{aligned}$$

$$\begin{aligned} \tan\delta_{\mu}=\frac{\mu^{''}}{\mu^{'}}\#\left( 6 \right) \end{aligned}$$

where *ε* describes the phase lag of a material's internal electric induction field (D) relative to the external field, *μ* describes the phase lag of its magnetic induction field (B) relative to the external field. The loss tangent represents the ability of a material to absorb electromagnetic wave energy and convert it into heat. A higher value of the loss tangent indicates a stronger ability of the material to absorb electromagnetic wave.

***S1.3.2 Cole-Cole Semicircle***

When the frequency of the alternating electric field increases to a certain extent, the polarization process cannot synchronize with the electric field, resulting in a phase difference between the electric field and polarization. As the frequency of the alternating electric field further rises to a specific level, it leads to the loss of electric field energy, namely the polarization relaxation phenomenon. The relaxation of dipole polarization can be described by Debye theory, and the real and imaginary parts of the complex permittivity can be expressed by Debye theory as follows:

$$\begin{aligned} \varepsilon^{'}=\varepsilon_{\infty}+\frac{\varepsilon_{s}-\varepsilon_{\infty}}{1+\omega^{2}\tau^{2}}\#\left( 7 \right) \end{aligned}$$

$$\begin{aligned} \varepsilon^{''}=\frac{\left( \varepsilon_{s}-\varepsilon_{\infty} \right)\omega\tau}{1+\omega^{2}\tau^{2}}\#\left( 8 \right) \end{aligned}$$

$$\begin{aligned} \tan\delta_{\varepsilon}=\frac{\varepsilon^{'}}{\varepsilon^{''}}=\frac{\left( \varepsilon_{s}-\varepsilon_{\infty} \right)\omega\tau}{\varepsilon_{s}+{\varepsilon_{\infty}\omega}^{2}\tau^{2}}\#\left( 9 \right) \end{aligned}$$

where *ε_s_* denotes the static permittivity, *ε_∞_* represents the high-frequency (optical) permittivity, *ω* is the angular frequency, and *τ* stands for the relaxation time. According to the above formula, the real part of the permittivity decreases with increasing frequency, while the imaginary part reaches a peak when *ωτ* = 1. This phenomenon of frequency-dependent permittivity is referred to as dielectric dispersion. When plotting the real part of the permittivity on the horizontal axis and the imaginary part on the vertical axis (Cole-Cole plot), the above equation can be rearranged to obtain:

$$\begin{aligned} {\left( \varepsilon^{'}-\frac{\varepsilon_{s}+\varepsilon_{\infty}}{2} \right)^{2}+\left( \varepsilon^{''} \right)}^{2}=\left( \frac{\varepsilon_{s}-\varepsilon_{\infty}}{2} \right)^{2}\#\left( 10 \right) \end{aligned}$$

The simplified form is obtained as follows:

$$\begin{aligned} \varepsilon^{'}=\varepsilon_{\infty}+\frac{1}{\tau}\left( \frac{\varepsilon^{''}}{\omega} \right)\#\left( 11 \right) \end{aligned}$$

It can be inferred that the dielectric polarization process manifests as semicircles in the Cole-Cole plot.

***S1.3.3 Impedance Matching Property***

The surface impedance of the material is jointly determined by the complex permittivity and complex permeability, which can be expressed as *Z*. The impedance matching characteristic serves to evaluate the ability of electromagnetic wave to penetrate into absorbing materials, which essentially reflects the matching relationship between the absorption and reflection of electromagnetic wave. The impedance matching property is typically denoted by "*Z*". It is jointly determined by the complex permittivity and complex permeability, and can be quantified by calculating the ratio *|**Z_in_/Z_0_|*. The specific calculation formula is as follows:

$$\begin{aligned} Z=\frac{Z_{in}}{Z_{0}}=\sqrt{\frac{\mu_{r}}{\varepsilon_{r}}\tan h\left( j\frac{2\pi fd}{c}\sqrt{\mu_{r}\varepsilon_{r}} \right)} \#\left( 12 \right) \end{aligned}$$

where *Z_in_* represents the input characteristic impedance, and *Z_0_* denotes the free space impedance. *f* is the frequency of electromagnetic wave, *d* is the thickness of the absorbing material, and *c* is the speed of light. RL stands for reflection loss. *μ_0_* is the magnetic permeability in vacuum, with a value of 4π × 10^-7^ H/m, and *ε_0_* is the dielectric constant in vacuum, with a value of 8.854 × 10^-12^ F/m. *μ_r_* represents the relative magnetic permeability (*µ_r_*), and *ε_r_* represents the relative dielectric constant (*ε_r_*).

***S1.3.4 Reflection Loss***

Reflection loss (RL) is a widely adopted metric for assessing the electromagnetic wave absorption performance of materials. Leveraging the transmission line theory and the measured electromagnetic parameters, and in conjunction with the specified sample thickness, the RL value of the material can be computed. The detailed calculation formula is presented as follows:

$$\begin{aligned} RL=20\lg\left| \frac{Z_{in}-Z_{0}}{Z_{in}+Z_{0}} \right| \#\left( 13 \right) \end{aligned}$$

$$\begin{aligned} Z_{in}=Z_{0}\sqrt{\frac{\mu_{r}}{\varepsilon_{r}}\tan h\left( j\frac{2\pi fd}{c}\sqrt{\mu_{r}\varepsilon_{r}} \right)}\#\left( 14 \right) \end{aligned}$$

RL value of -10 dB implies that the absorbing material has absorbed 90% of the incident electromagnetic wave. When the RL value reaches -20 dB, it means that the material can achieve an electromagnetic wave loss rate of up to 99%. Evidently, as the RL value decreases, the loss rate of the material for electromagnetic wave increases correspondingly. Conventionally, a RL below -10 dB is defined as effective absorption, and the width of the corresponding frequency range is called the effective absorption bandwidth (EAB).

***S1.3.5 Characteristic Impedance***

In the frequency bands with a large attenuation constant, when the impedance matching coefficient *Z=|Z_in_/Z_0_|* is equal to or close to 1 (i.e., the two impedance values of the electromagnetic wave absorbing material are similar), the material can achieve ideal impedance matching and exhibit the optimal electromagnetic wave absorption performance.

***S1.3.6 Dielectric Loss***

The dielectric loss of electromagnetic wave absorbing materials includes conduction loss ($\varepsilon_{c}^{''}$) and polarization loss ($\varepsilon_{p}^{''}$), which can be simulated by connecting resistors and capacitors in series and parallel circuits. The $\varepsilon_{c}^{''}$ and $\varepsilon_{p}^{''}$ are calculated according to the formula:

$$\begin{aligned} \varepsilon^{''}=\varepsilon_{p}^{''}+\varepsilon_{c}^{''}=\frac{2\pi f\tau\left( \varepsilon_{s}-\varepsilon_{\infty} \right)}{1+\left( 2\pi f \right)^{2}\tau^{2}}+\frac{\sigma}{2\pi f\varepsilon_{0}}\#\left( 15 \right) \end{aligned}$$

$$\begin{aligned} \varepsilon_{c}^{''}=\frac{\sigma}{2\pi f\varepsilon_{0}}\#\left( 16 \right) \end{aligned}$$

where $\sigma$ is the conductivity, *τ* represents the polarization relaxation time, and *ε_0_* (8.85 × 10^-12^ F/m) denotes the vacuum permittivity.

**S1.4 Radar Cross Section Simulation**

The radar cross section (RCS) simulations were performed in CST software based on the far-field response. The simulation model consists of an upper wave-absorbing layer and a lower perfect electrical conductor (PEC) layer. The model was placed in the XOY plane, and a plane wave was incident along the negative Z direction. Open boundary conditions were applied to simulate the free-space environment. The RCS performance of the samples was evaluated by the following equation:

$$\begin{aligned} RCS\left( dBm^{2} \right)=10log\left( \frac{4\pi S}{\lambda^{2}}\left| \frac{E_{S}}{E_{i}} \right|^{2} \right)\#\left( 17 \right) \end{aligned}$$

where *S* is the area of the layer of the simulated plate, and *λ* is the electromagnetic wave wavelength. *E_s_* and *E_i_* represent the electric field intensity of scattered electromagnetic wave and incident electromagnetic wave, respectively.

**S2 Supplementary Detail**

**S2.1 RCS simulation**

The simulation model consisted of a 180 mm × 180 mm square absorbing layer deposited on a perfect electric conductor (PEC) substrate of the same lateral dimensions. The thicknesses of the PEG-MXene@CuS absorbing layer and the PEC substrate were 1.88 mm and 1.0 mm, respectively. The simulations were performed using the Integral Equation (IE) Solver in CST Studio Suite 2024. A plane wave was used as the excitation source under open boundary conditions. The azimuth angle was fixed at 90°, and the incident angle (θ) was varied from -60° to 60°. The simulations were conducted at a representative frequency of 14.9 GHz using the experimentally measured complex permittivity and permeability obtained by the coaxial-line method.

**S2.2 DFT calculation**

All the density-functional theory (DFT) computations were performed using the Cambridge Sequential Total Energy Package (CASTEP)^[1]^ based on the pseudopotential plane wave (PPW) method. Electron-ion interactions were described using the ultrasoft (USP) potentials^[2]^. A plane-wave basis set was employed to expand the wave functions with a cutoff kinetic energy of 400 eV. For the electron-electron exchange and correlation interactions, the functional parametrized by Perdew-Burke-Ernzerhof (PBE)^[3]^, a form of the general gradient approximation (GGA), was used throughout with the Hubbard U values of Cu is 4.5 eV and 2.5 eV for Ti. The vander Waals interaction was described using the DFT-D2 method that proposed by Grimme^[4]^.

During the geometry optimizations, all the atom position were allowed to relax. In this work, the Brillouin-zone integrations were conducted using Monkhorst-Pack (MP) grids^[5]^ of special points with the separation of 0.05 Å^-1^ for the model cell. The convergence criterion for the electronic self-consistent field (SCF) loop was set to 1×10^-6^ eV/atom. The atomic structures were optimized until the residual forces were below 0.03 eVÅ^-1^.

**S3 Supplementary Figure**

**
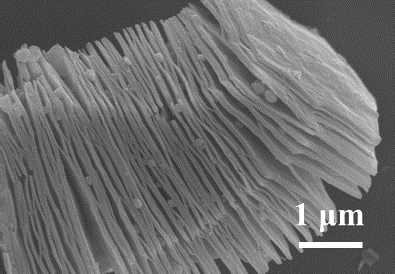
**

Fig. S1 SEM image of MXene

**
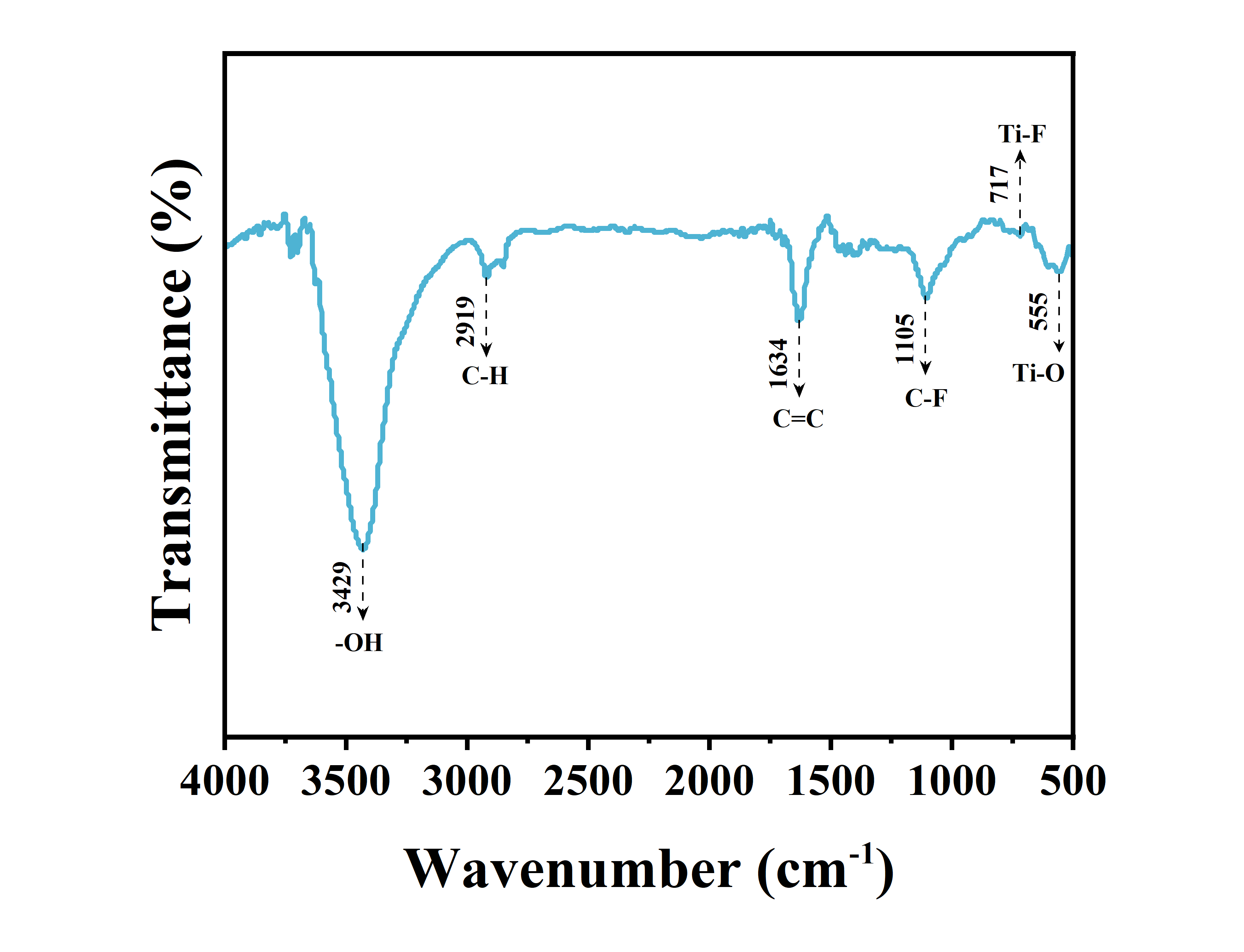
**

Fig. S2 FT-IR spectra of MXene^[6]^


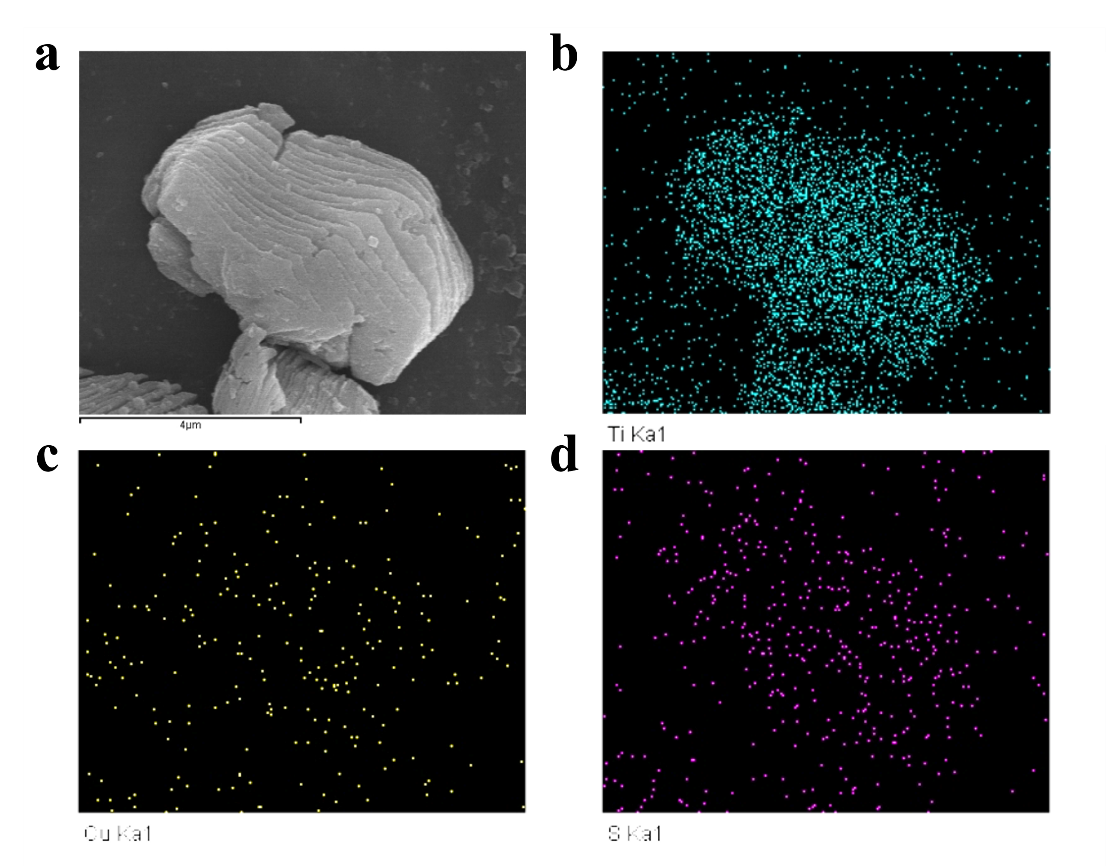


**Fig. S3** EDS elemental mapping of MXene@CuS-1


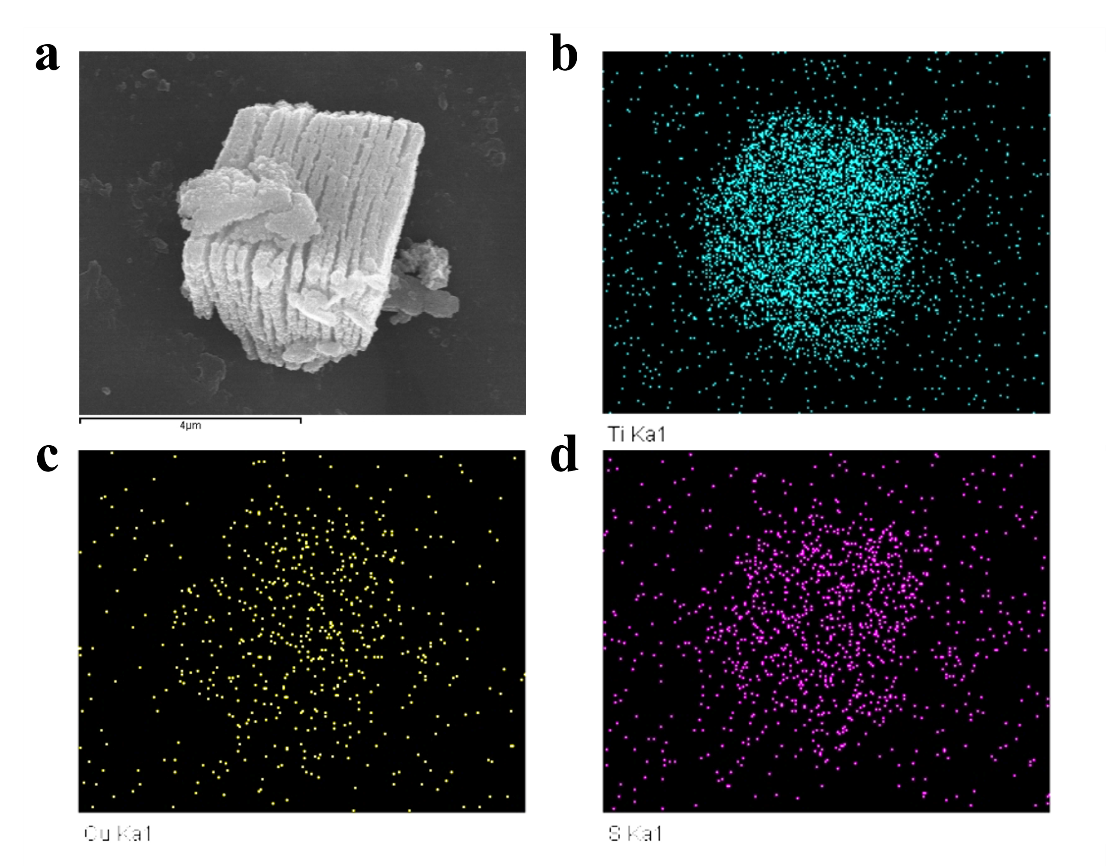


**Fig. S4** EDS elemental mapping of MXene@CuS-2


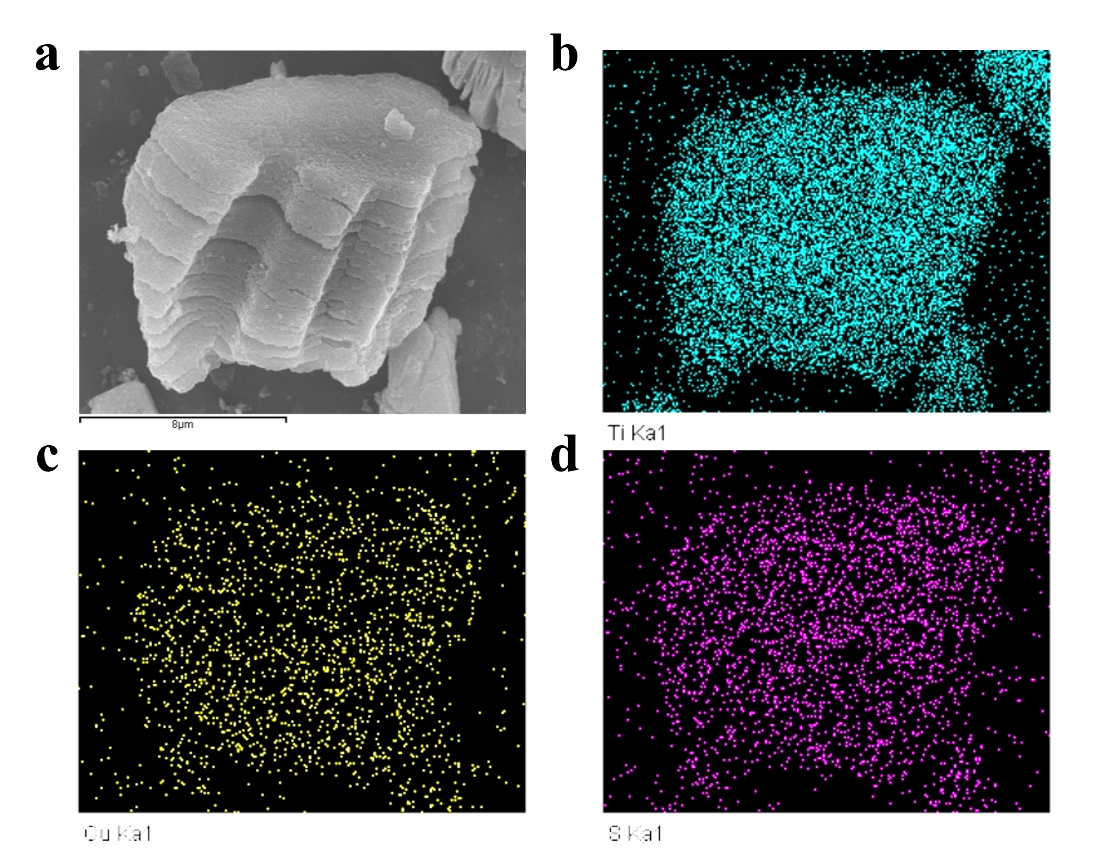


**Fig. S5** EDS elemental mapping of MXene@CuS-3

**
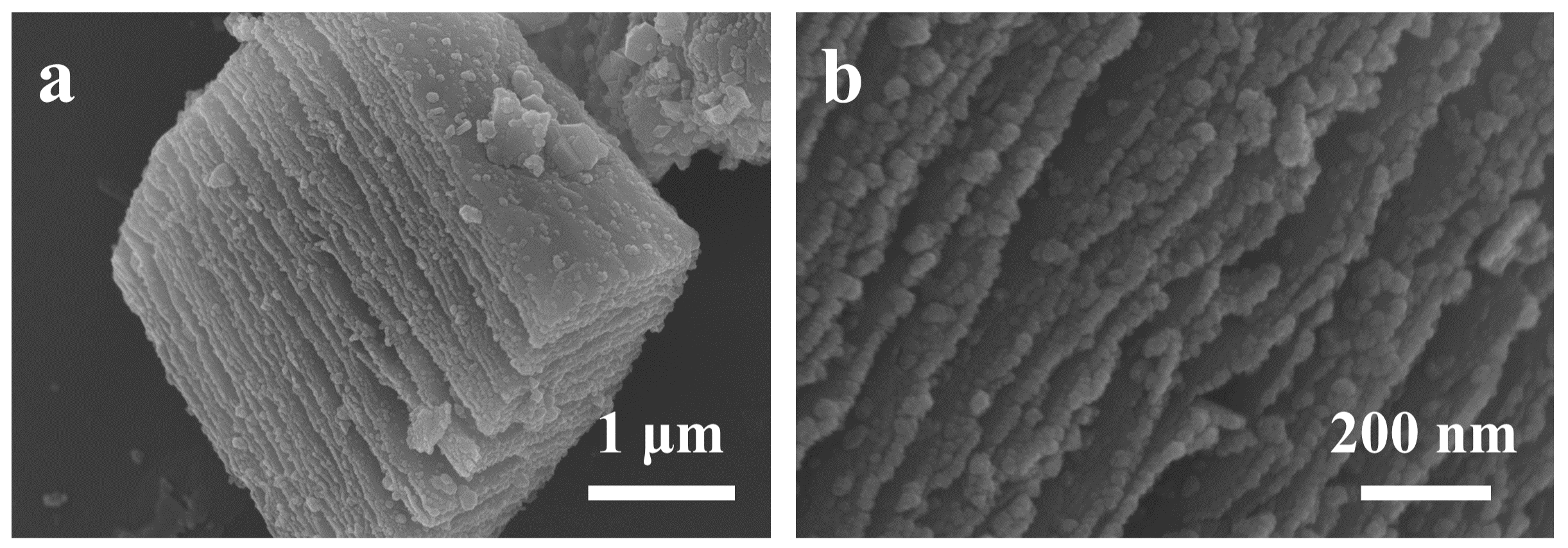
**

Fig. S6 SEM images of MXene@CuS-1

**
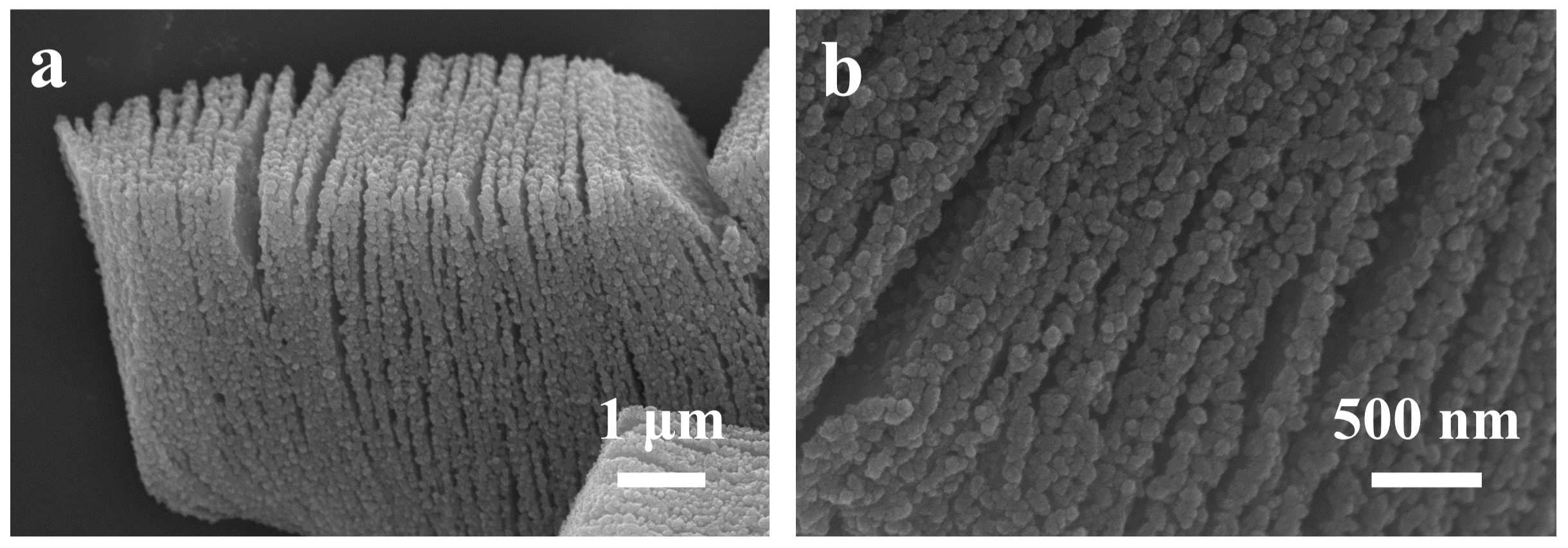
**

Fig. S7 SEM images of MXene@CuS-2

**
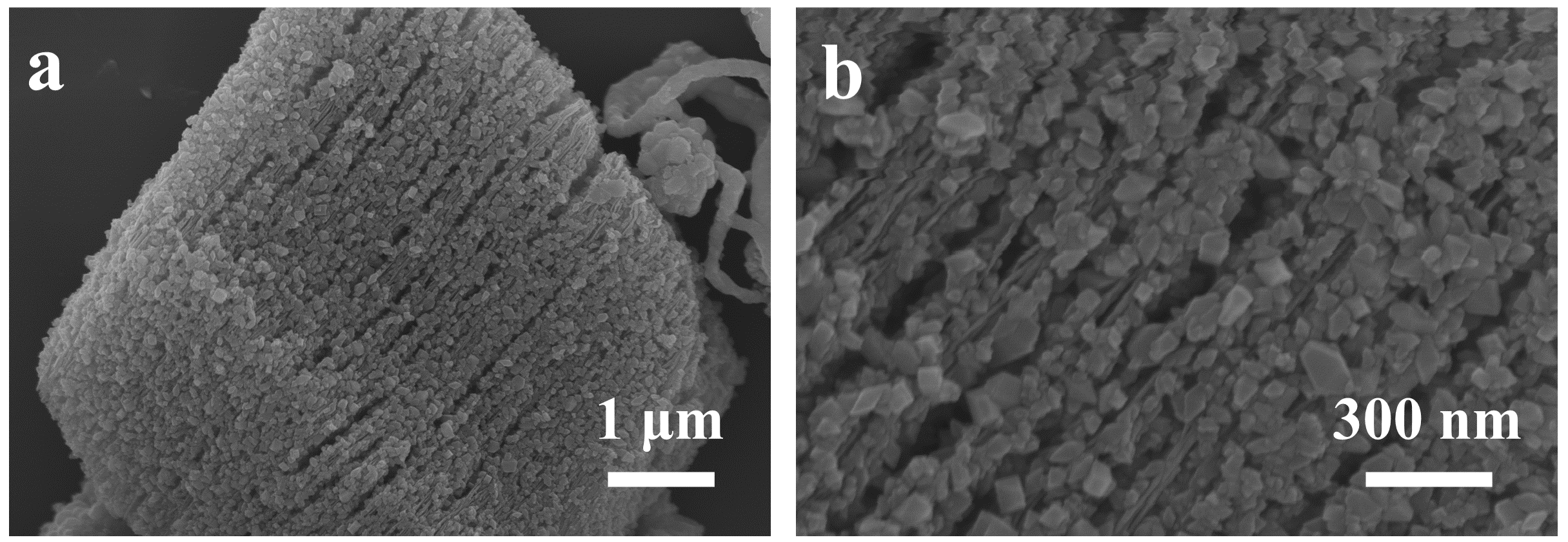
**

Fig. S8 SEM images of MXene@CuS-3


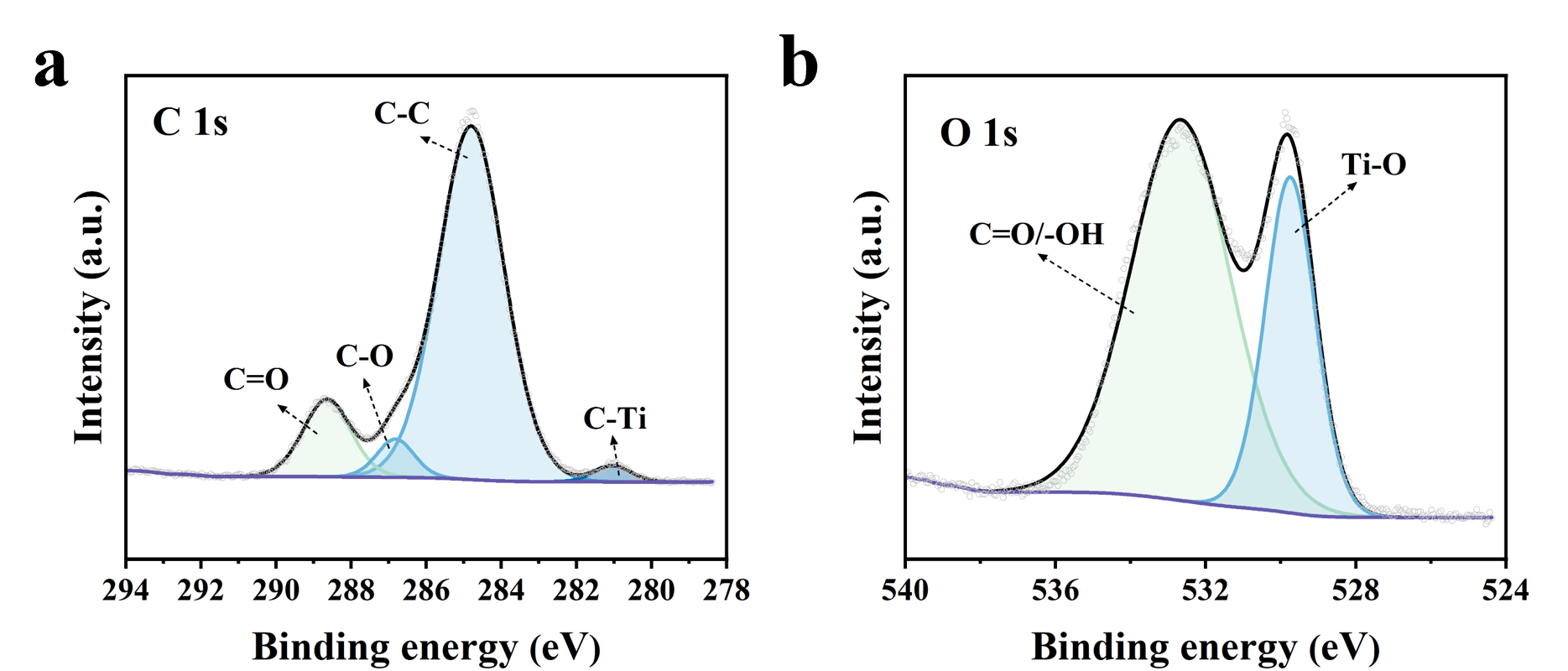


**Fig. S9** XPS spectra of MXene@CuS: **a** C 1s, **b** O 1s

**
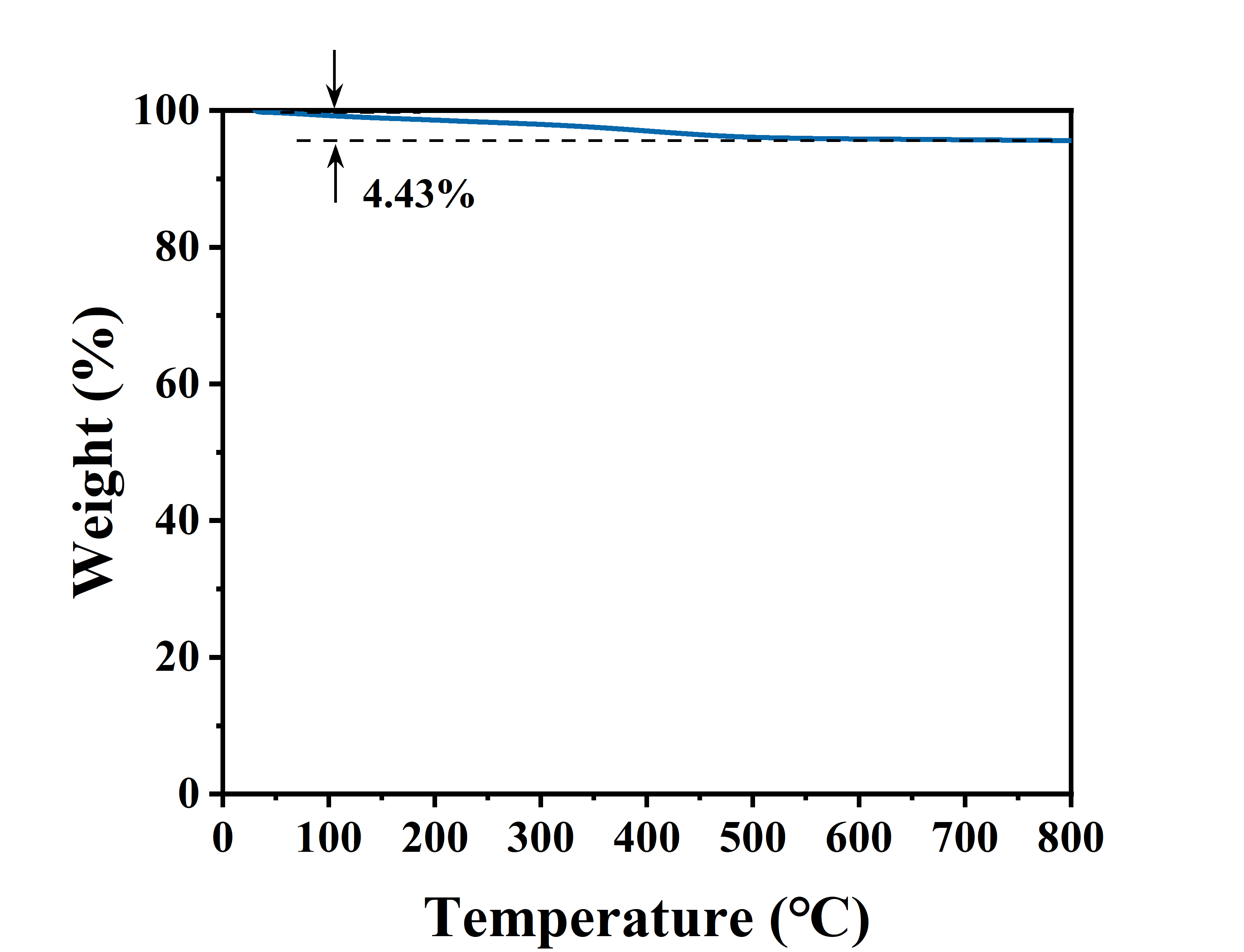
**

Fig. S10 TGA curves of MXene@CuS

**
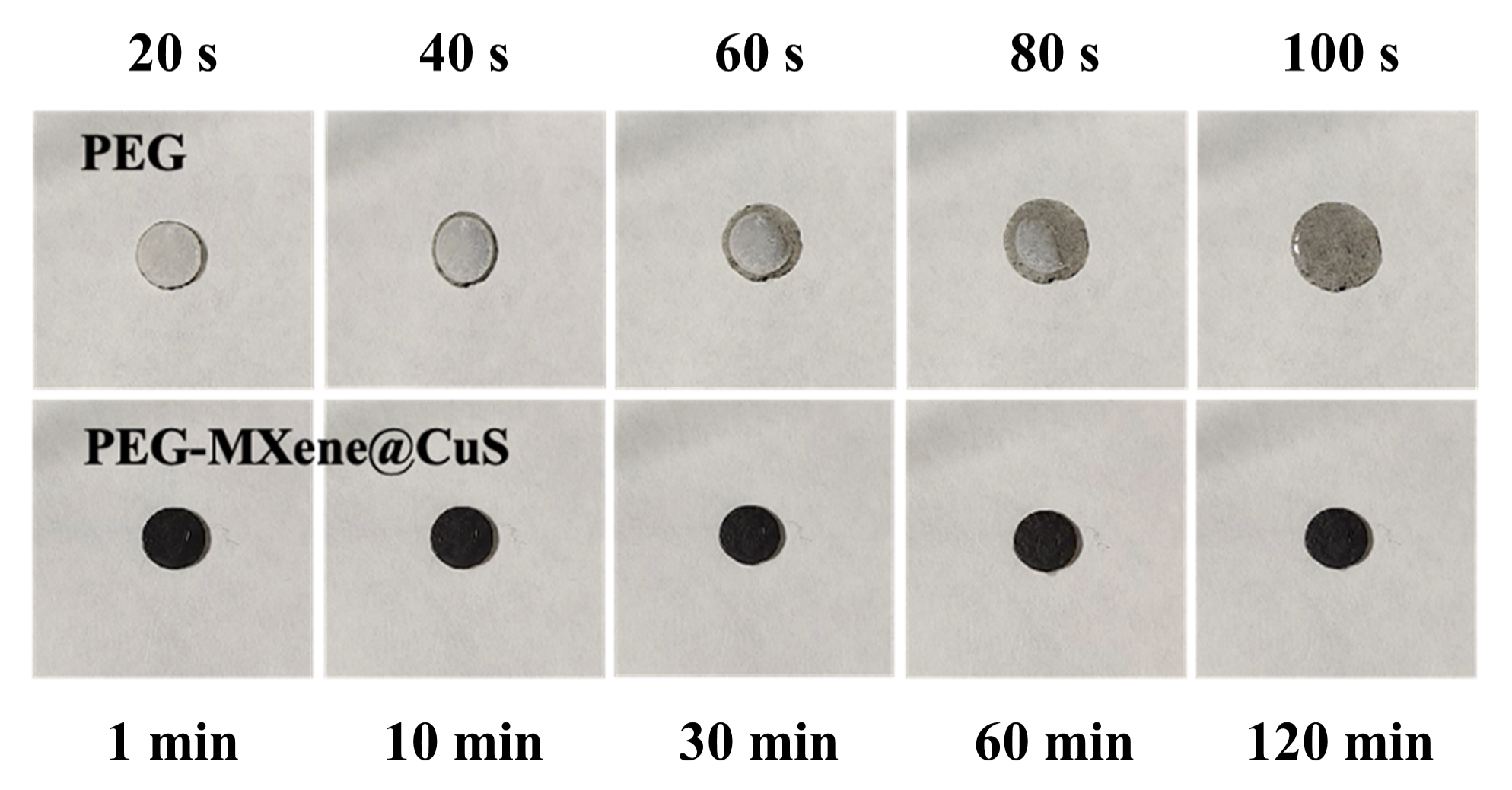
**

**Fig. S11** Leakage test experiment of PEG and PEG-MXene@CuS

**
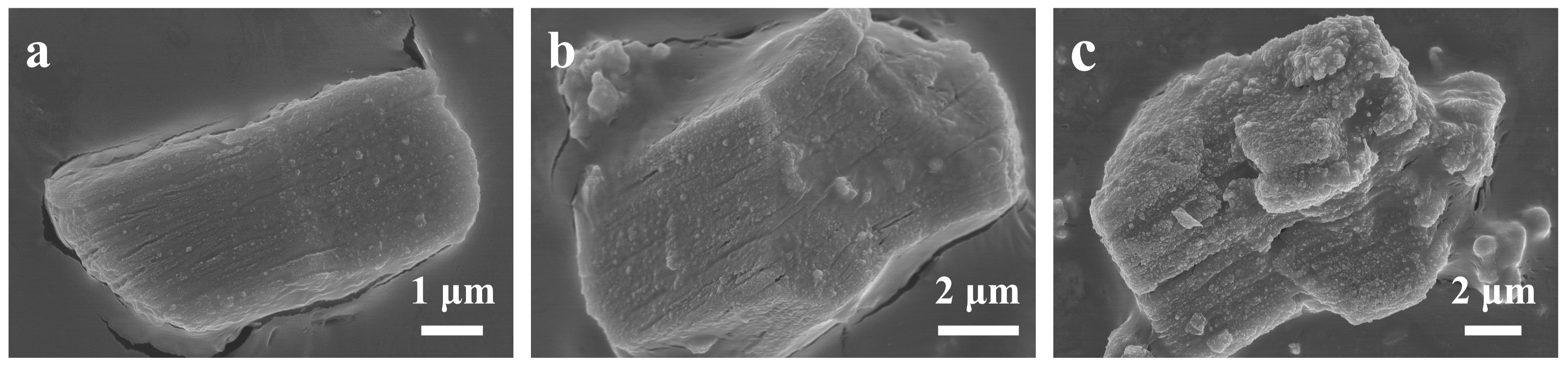
**

Fig. S12 SEM images of a PEG-MXene@CuS-1, b PEG-MXene@CuS-2, and c PEG-MXene@CuS-3

**
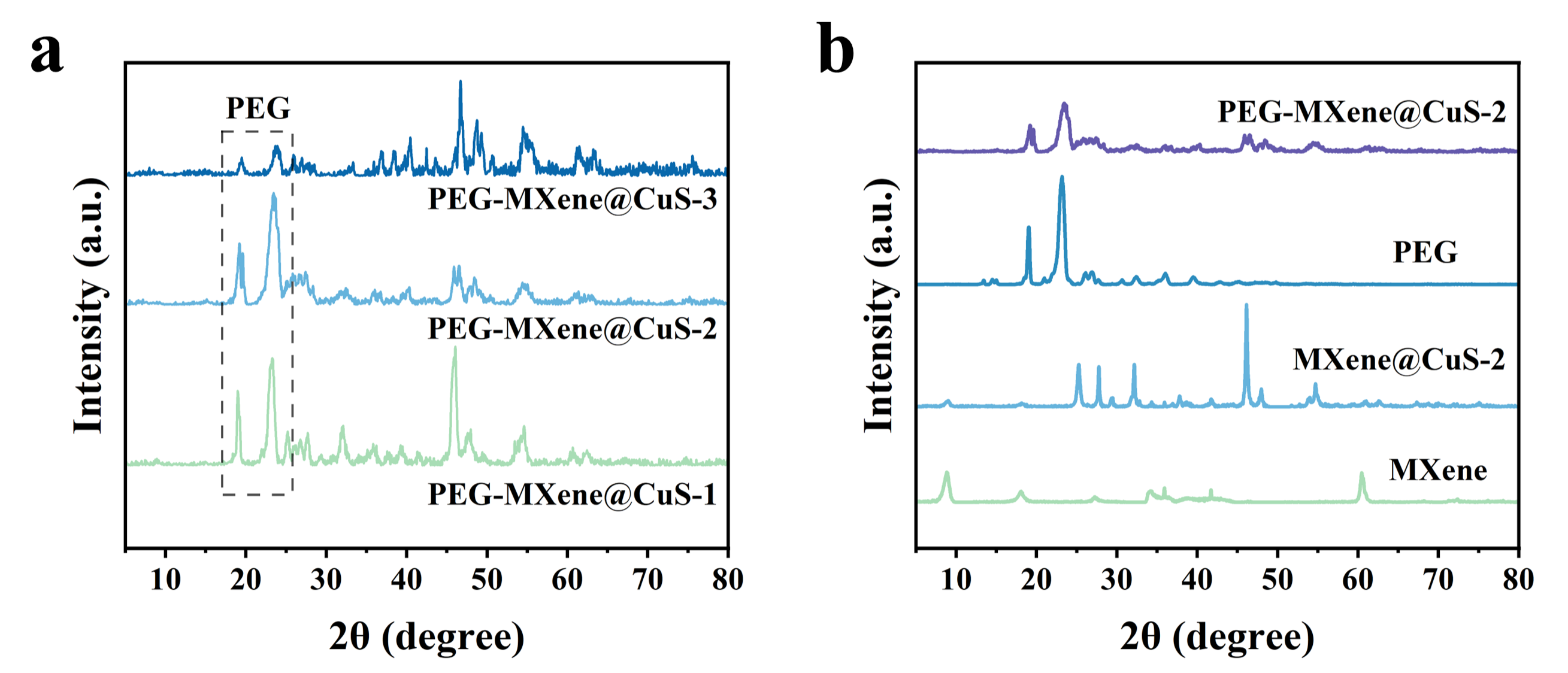
**

**Fig. S13** **a** XRD patterns of PEG-MXene@CuS. **b** XRD patterns of MXene, MXene@CuS-2, PEG, and PEG-MXene@CuS-2

**
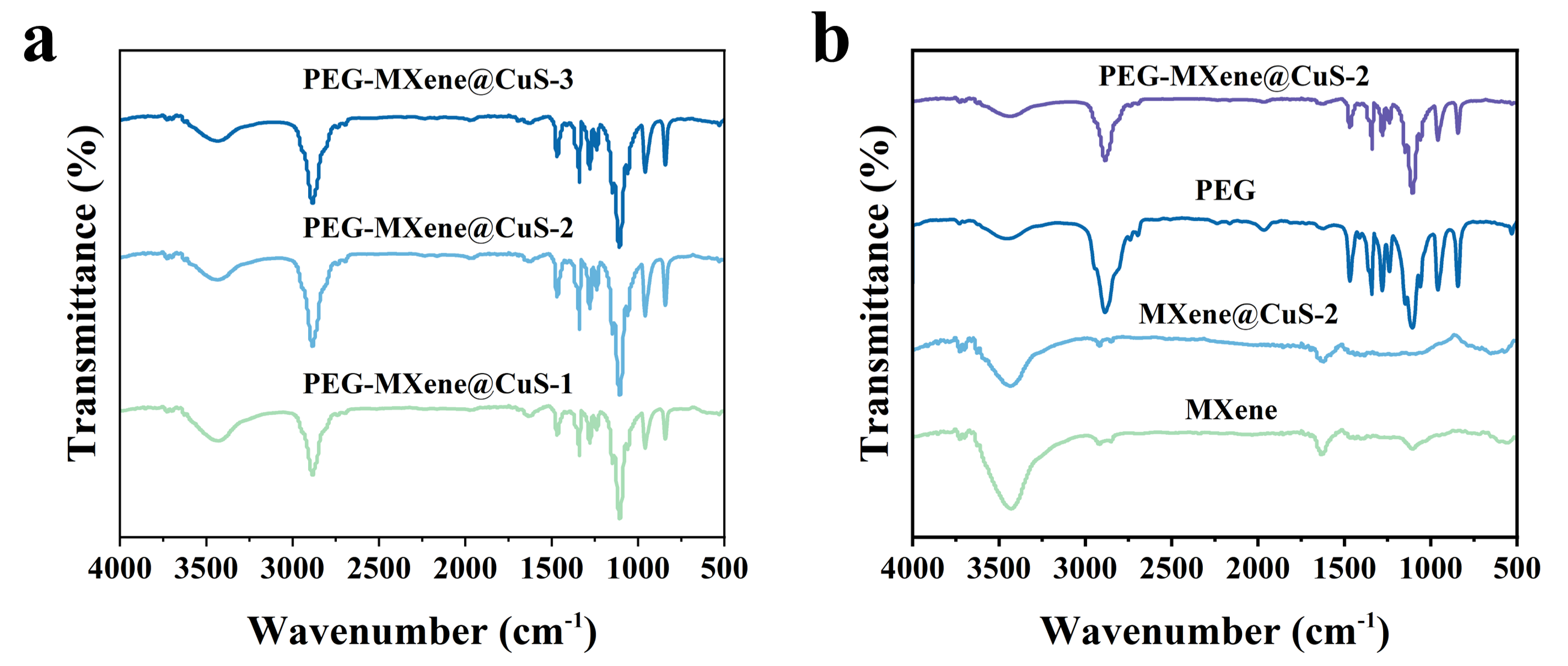
**

**Fig. S14** **a** FT-IR spectra of PEG-MXene@CuS. **b** FT-IR spectra of MXene, MXene@CuS-2, PEG, and PEG-MXene@CuS-2

**
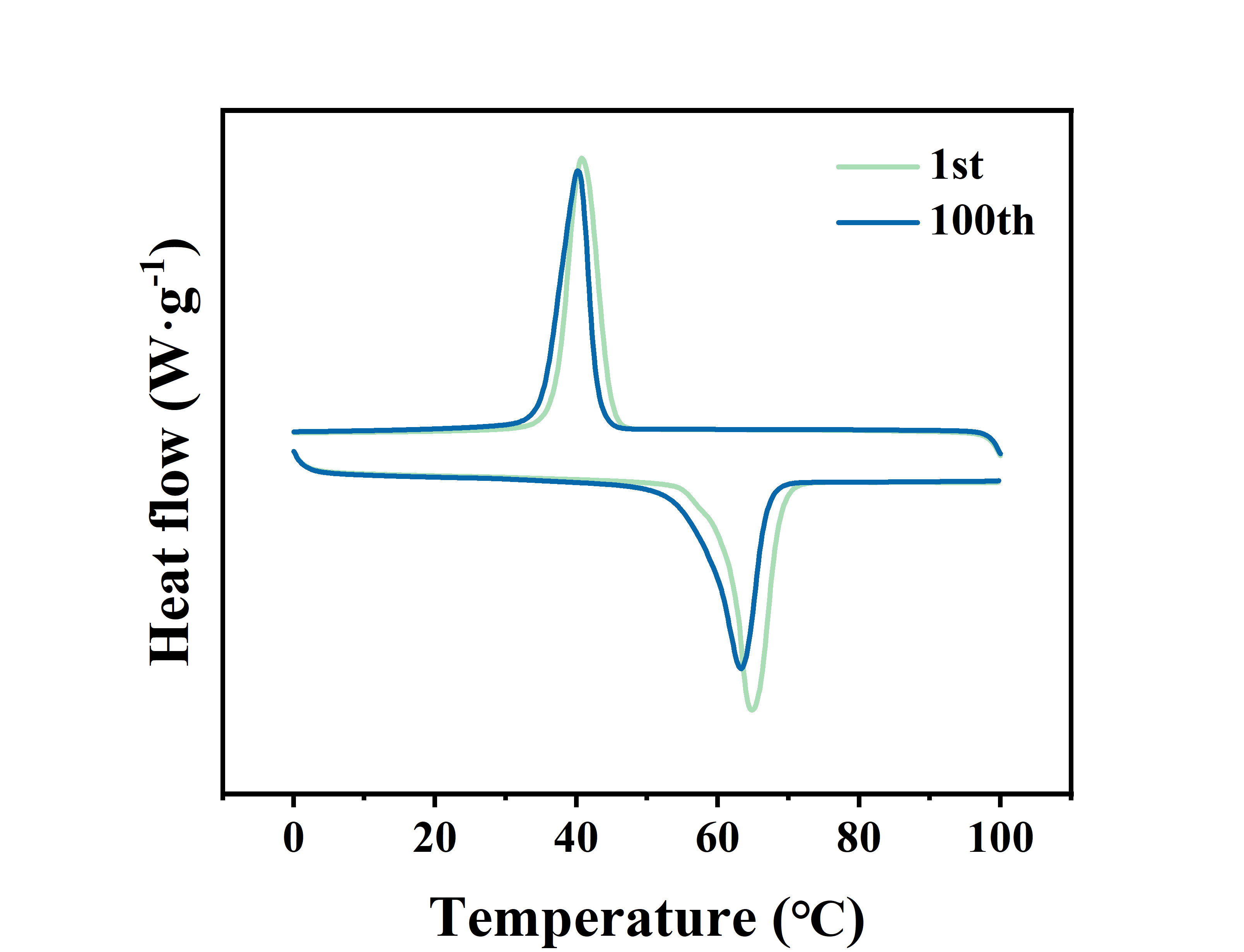
**

**Fig. S15** DSC curve of PEG-MXene@CuS-2 for the 1st and the 100th cycles

**
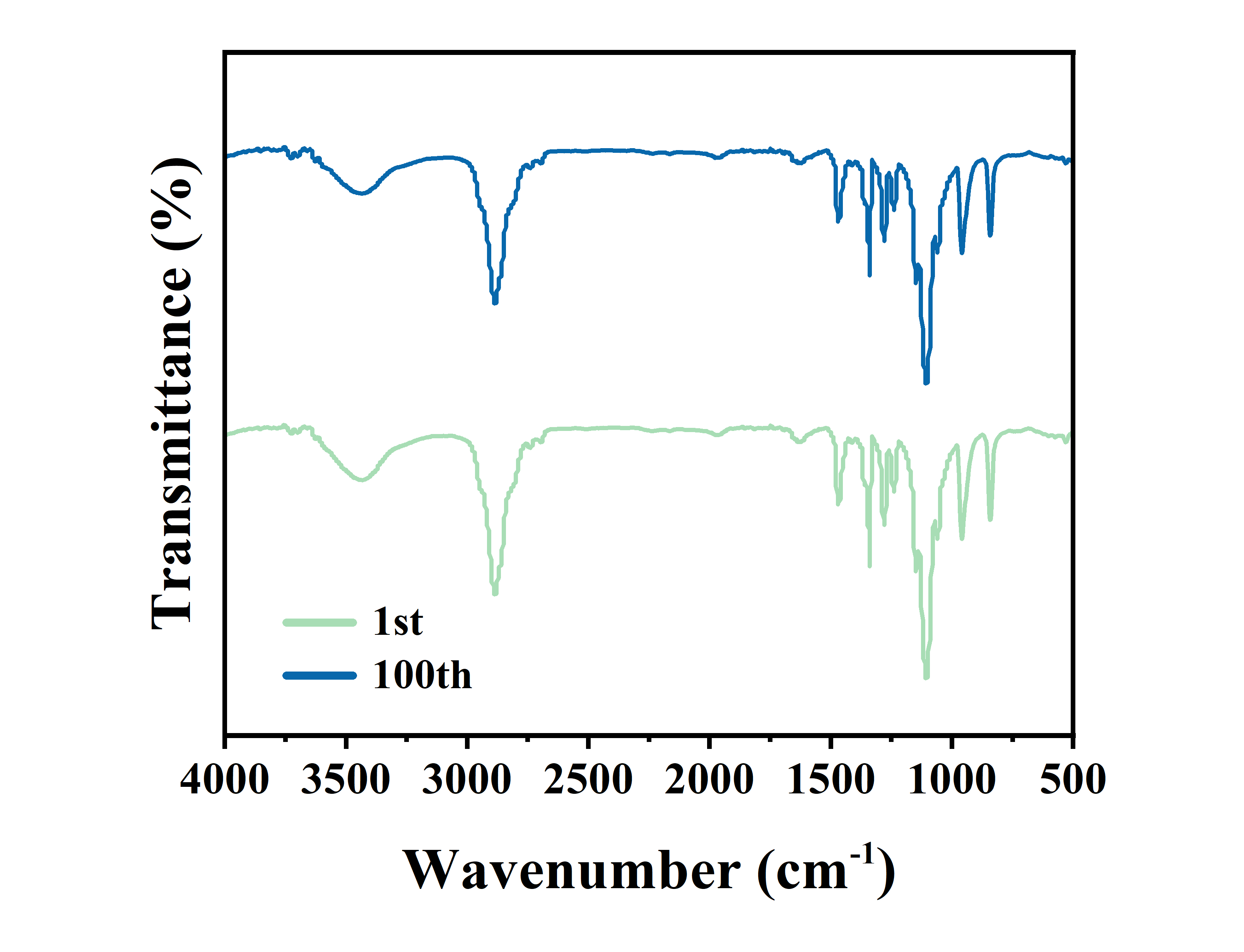
**

**Fig. S16** FT-IR spectra of PEG-MXene@CuS-2 for the 1st and the 100th cycles

**
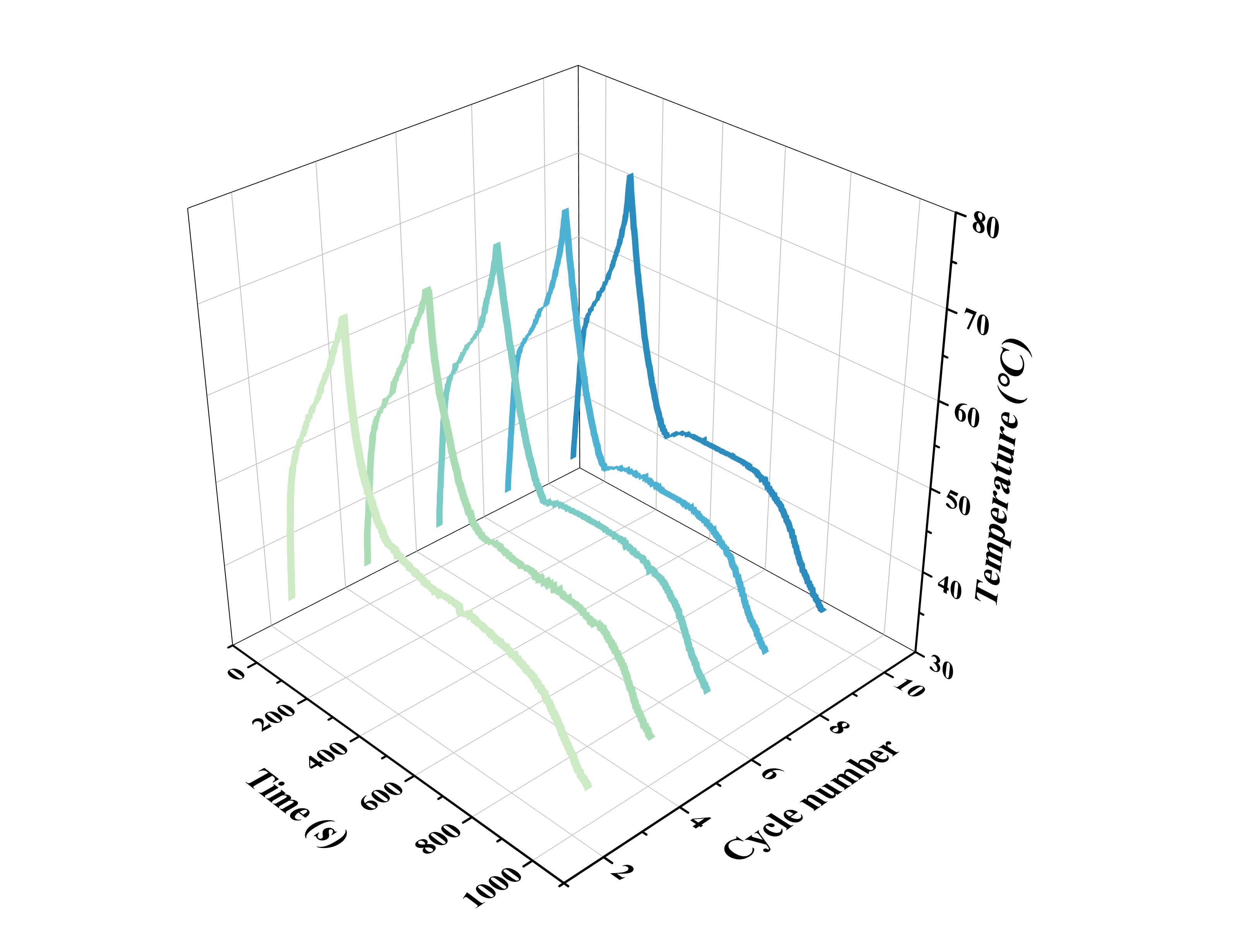
**

**Fig. S17** Cyclic photothermal conversion curves of PEG-MXene@CuS-2


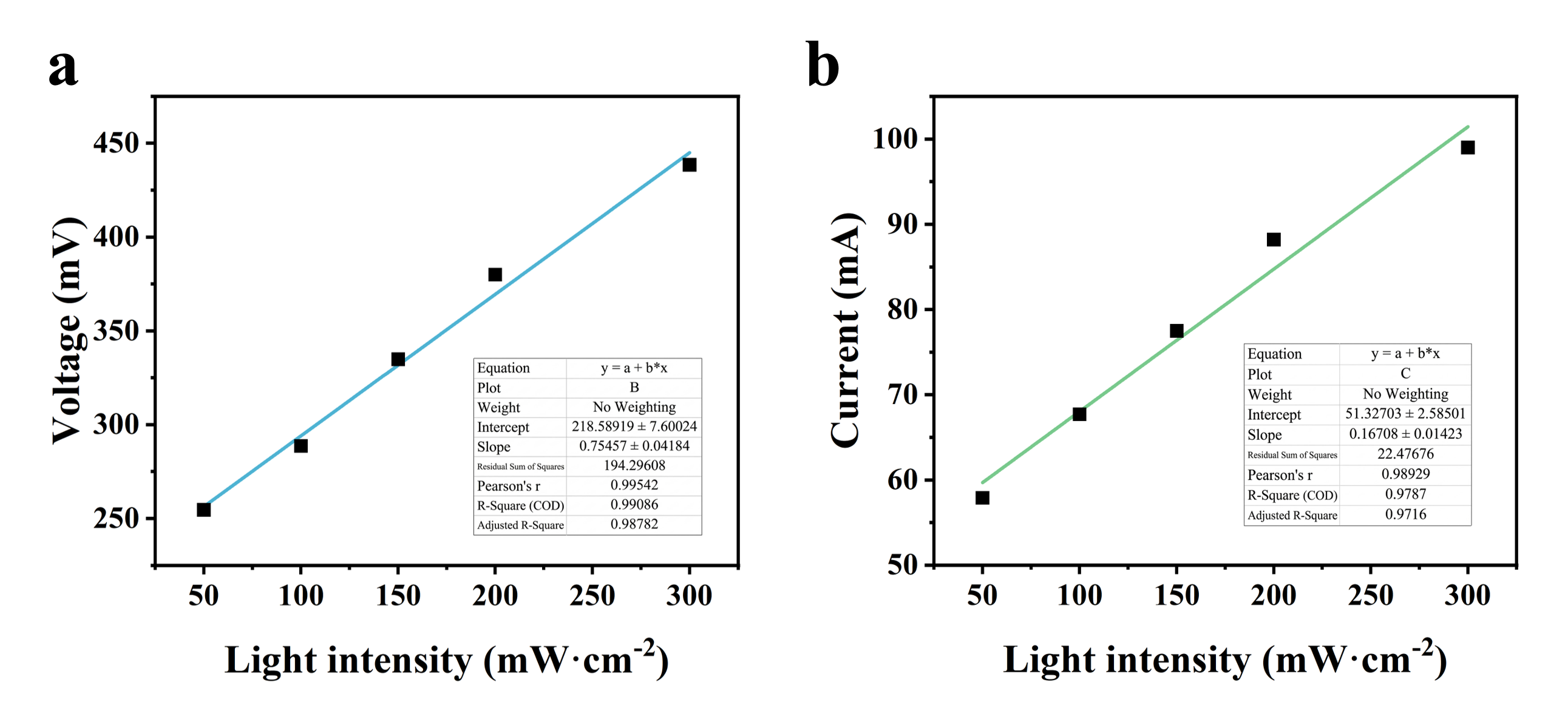


**Fig. S18** Fitting curves of the output voltage and current of PEG-MXene@CuS-2 varying irradiation intensities


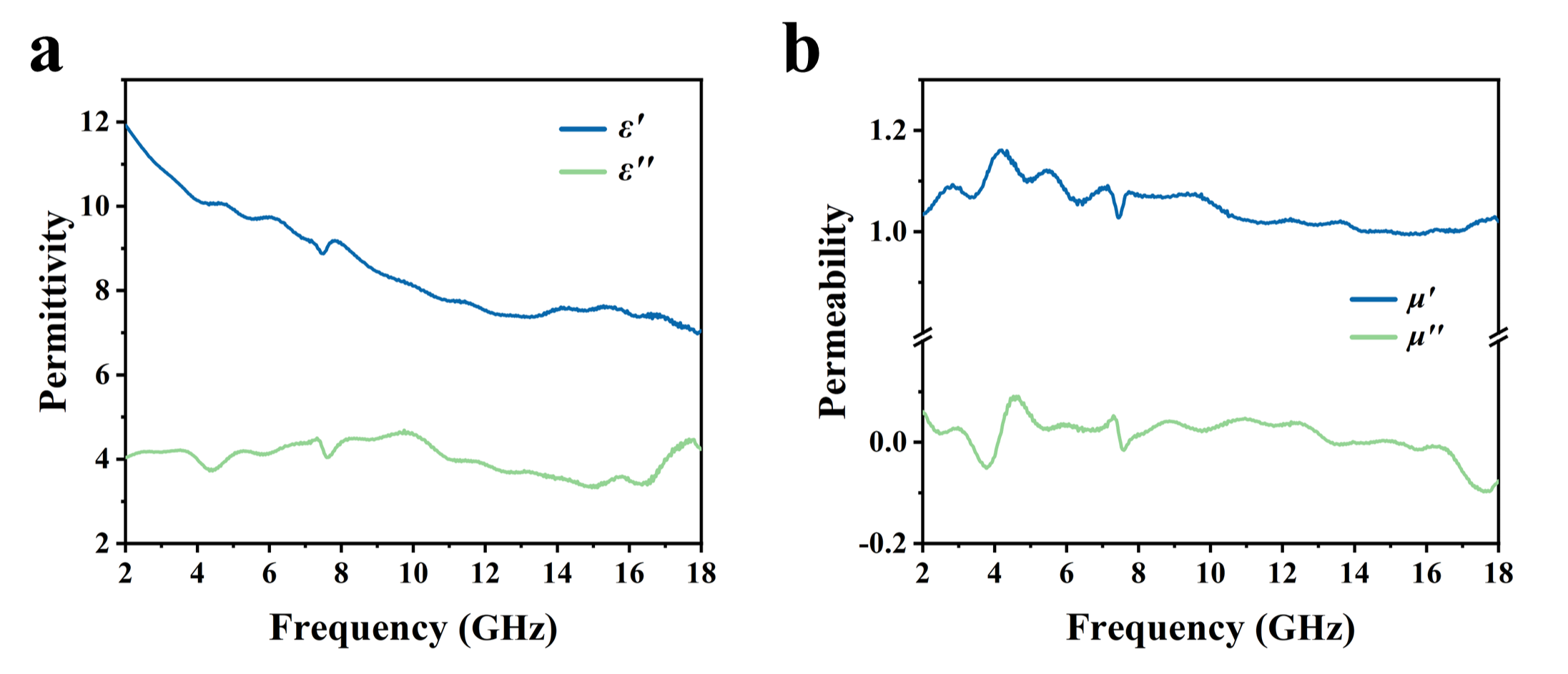


**Fig. S19** Electromagnetic parameters **a** *εʹ*, *εʺ* and **b** *μʹ*, *μʺ* of PEG-MXene@CuS-2

**
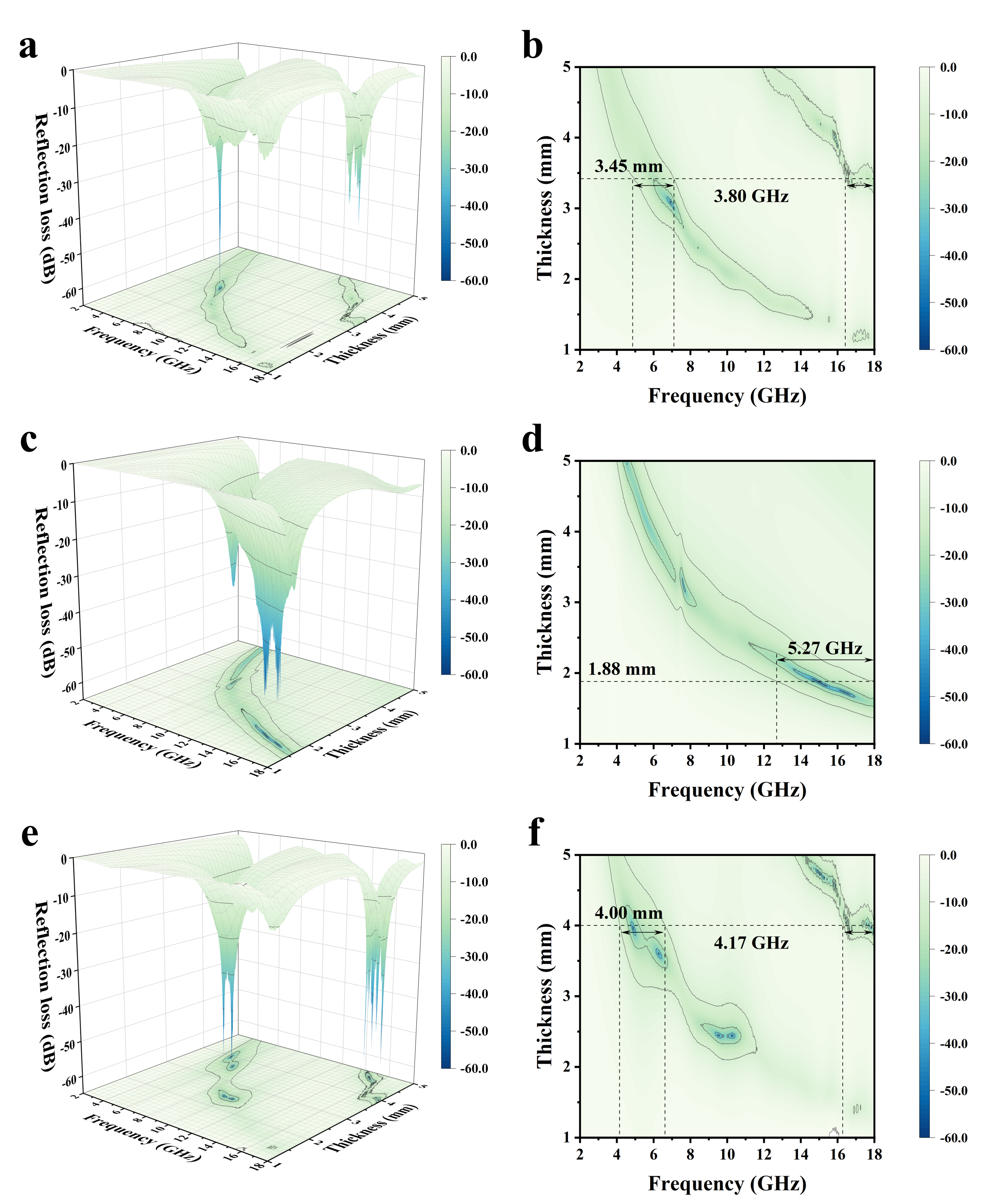
**

**Fig. S20** 3D representations and 2D projection graphics of the RL value for **a**, **b** PEG-MXene@CuS-1; **c**, **d** PEG-MXene@CuS-2; **e**, **f** PEG-MXene@CuS-3

**
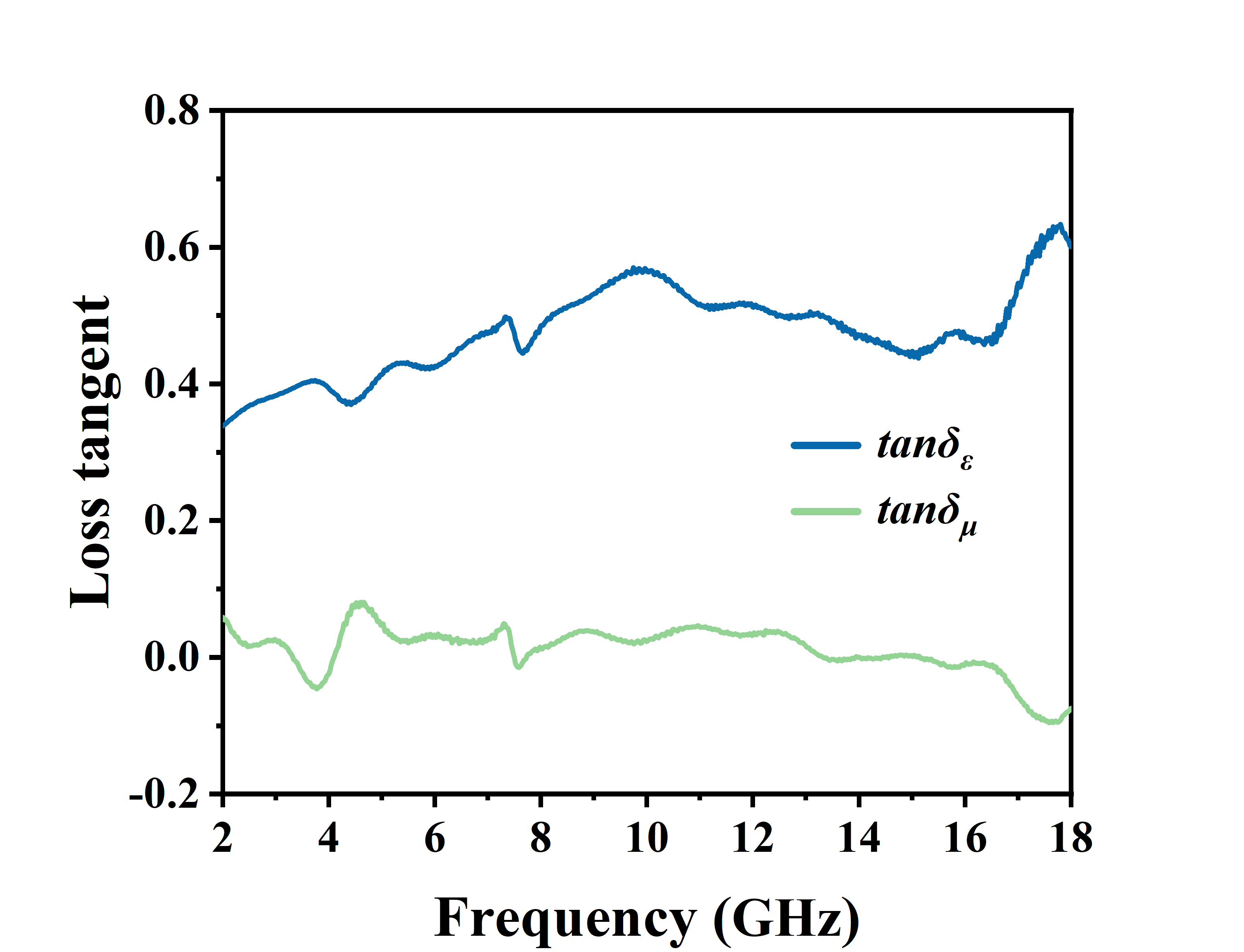
**

**Fig. S21** *tanδ_ε_* and *tanδ_μ_* of PEG-MXene@CuS-2


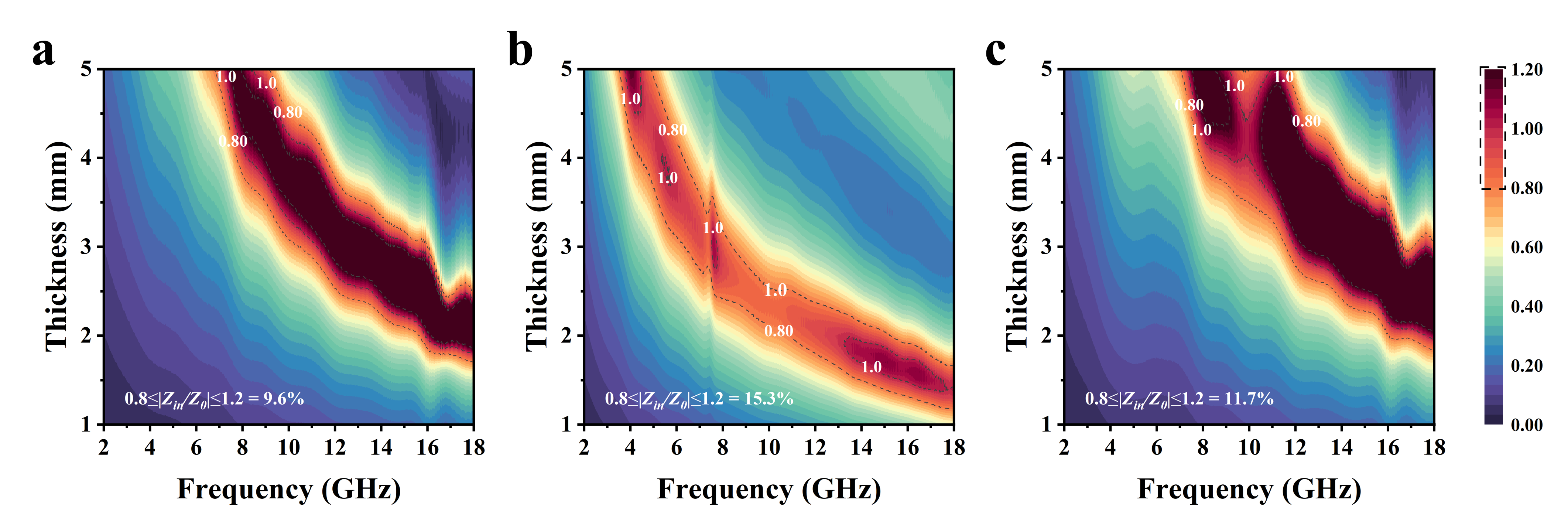


**Fig. S22** Impedance matching diagram of **a** PEG-MXene@CuS-1, **b** PEG-MXene@CuS-2, **c** PEG-MXene@CuS-3


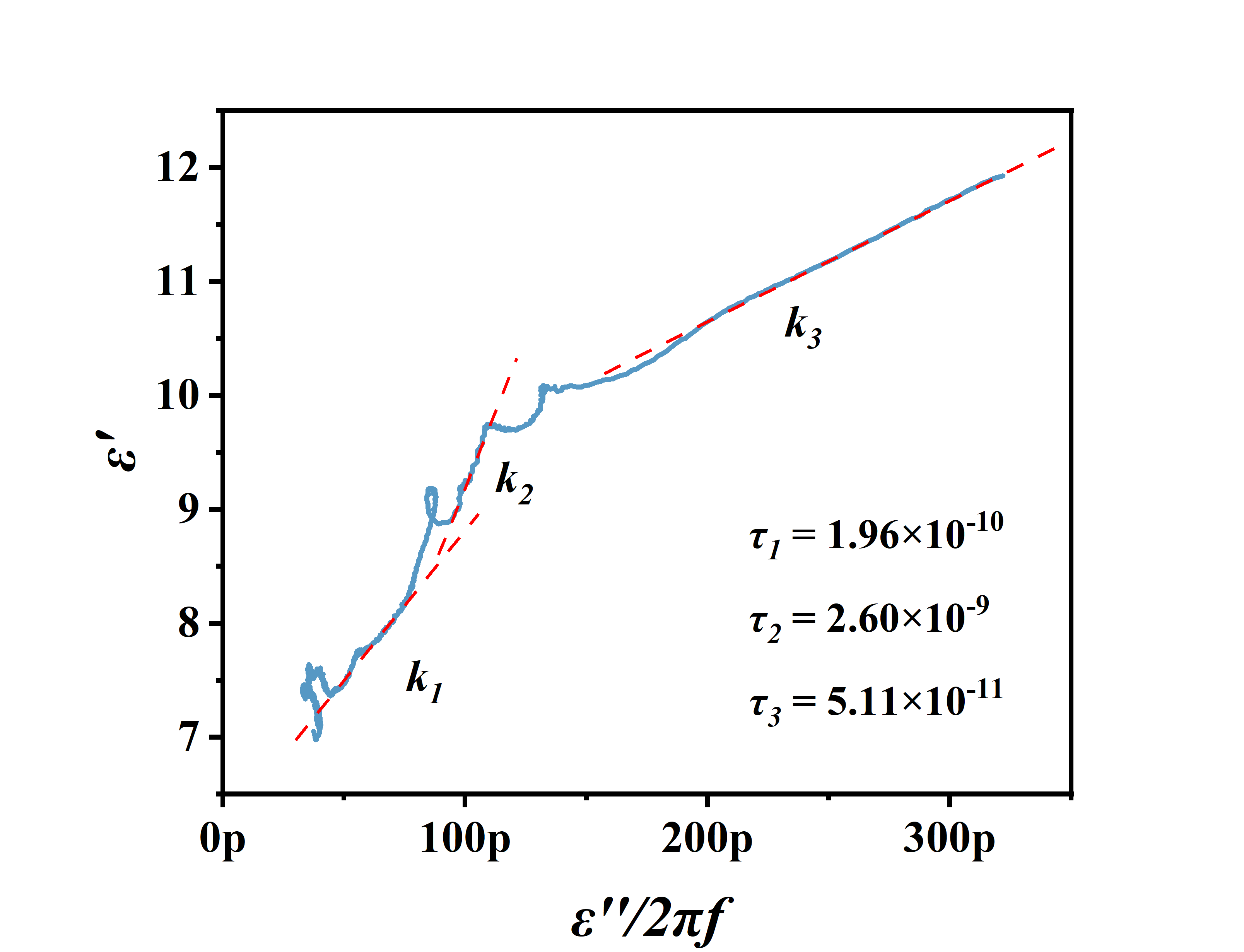


**Fig. S23** Linear fitting of *ε'* versus *εʹ/2πf* based on the Debye relaxation model


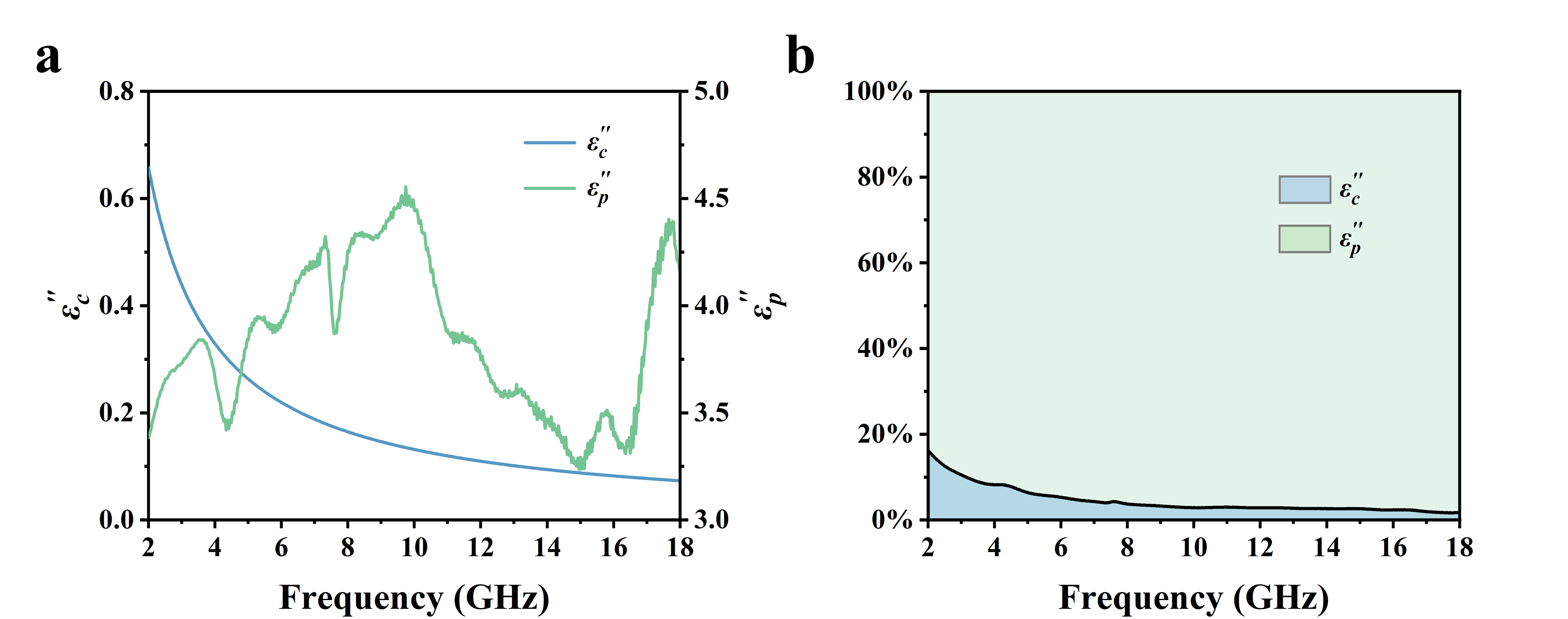


**Fig. S24 a** Conduction loss and polarization loss of PEG-MXene@CuS-2. **b** Ratio of conduction loss and polarization loss


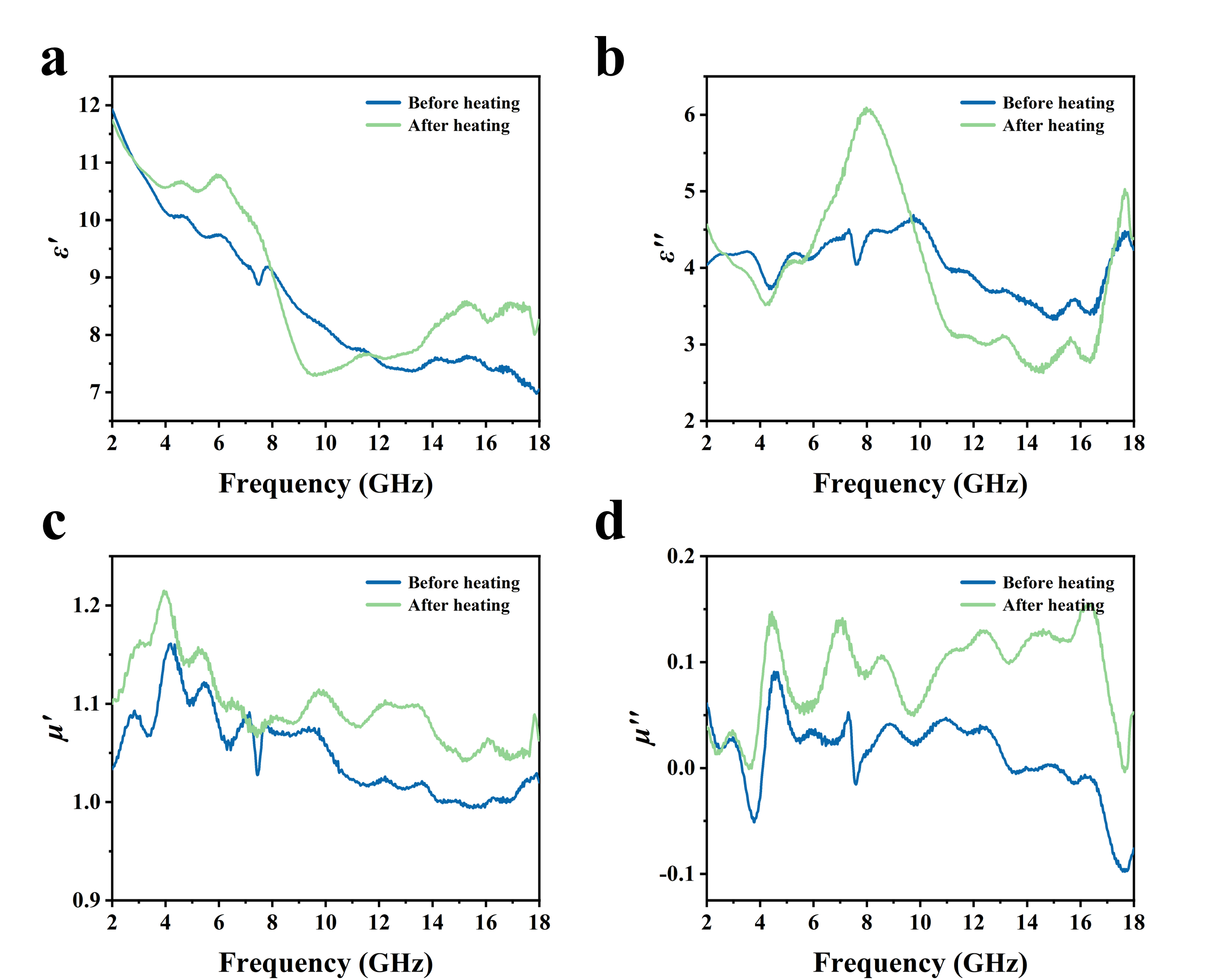


**Fig. S25** Electromagnetic parameters **a** *εʹ*, **b** *εʺ*, **c** *μʹ*, and **d** *μʺ* of PEG-MXene@CuS-2 before and after heating

**S4 Supplementary Table**

**Table S1** Elemental composition (Atomic %) of MXene@CuS samples

| Sample | C | Ti | Cu | S |
| --- | --- | --- | --- | --- |
| MXene@CuS-1 | 73.12% | 20.45% | 3.46% | 2.97% |
| MXene@CuS-2 | 65.46% | 25.57% | 4.89% | 4.09% |
| MXene@CuS-3 | 50.70% | 32.23% | 8.85% | 8.22% |

**Table S2** Thermal characteristics of PEG and composite PCMs

| Sample | Melting process | | Crystallization process | |
| --- | --- | --- | --- | --- |
|  | T_m_ (℃) | H_m_ (J·g^-1^) | T_c_ (℃) | H_c_ (J·g^-1^) |
| PEG | 61.1 | 152.83 | 44.6 | 140.07 |
| PEG-MXene@CuS-1 | 61.6 | 100.74 | 45.4 | 99.57 |
| PEG-MXene@CuS-2 | 60.2 | 99.06 | 45.2 | 99.36 |
| PEG-MXene@CuS-3 | 60.2 | 98.10 | 46.2 | 97.41 |

**Table S3** Thermal conductivity

| Sample | Thermal conductivity (W·m^-1^·K^-1^) |
| --- | --- |
| PEG | 0.25 |
| PEG-MXene | 0.41 |
| PEG-MXene@CuS-1 | 0.56 |
| PEG-MXene@CuS-2 | 0.68 |
| PEG-MXene@CuS-3 | 0.62 |

**Table S4** Photothermal conversion efficiency of PEG-MXene@CuS

| Sample | Photothermal conversion efficiency |
| --- | --- |
| PEG-MXene@CuS-1 | 89.6% |
| PEG-MXene@CuS-2 | 94.5% |
| PEG-MXene@CuS-3 | 91.1% |

**Table S5** Comparison of output voltage and photothermal efficiency for composite PCMs

| Sample | Output voltage (mV) | Photothermal efficiency | Light intensity (mW·cm^-2^) | Refs. |
| --- | --- | --- | --- | --- |
| TRIS/EG | 93.1 | 87.0% | 47.7 | S7 |
| PW-CNT/rGO | 150.0 | 90.3% | 150 | S8 |
| SA-BC/graphene | 151.9 | 90.8% | 200 | S9 |
| TD-ACW/Fe_3_O_4_ | 196.6 | 89.8% | 150 | S10 |
| PEG-PI/KNF/CoFe_2_O_4_/PPy | 259.7 | 84.8% | 200 | S11 |
| PEG-PPy | 318.0 | 86.0% | 250 | S12 |
| PW-PVP/GO/AgNPs | 200.5 | 92.6% | 100 | S13 |
| PEG-PU/NR/MXene | 410.0 | 92.0% | 200 | S14 |
| PEG-MoS_2_/Mt | 458.0 | 96.5% | 200 | S15 |
| PEG-PVA/HNT/PPy | 587.0 | 90.4% | 300 | S16 |
| PEG-BC/MoS_2_/CuS | 157.0 | 93.6% | 100 | S17 |
| PEG-CKF@Co/C | 309.0 | 94.4% | 100 | S18 |
| PEG-MXene@CuS | 288.6 | 94.5% | 100 | This work |

**Table S6** Photoresponsive thermoelectric performance of PEG-MXene@CuS-2

| Irradiation (mW·cm^-2^) | Output voltage (mV) | Output current (mA) | Power density (W·m^-2^) |
| --- | --- | --- | --- |
| 50 | 254.6 | 57.9 | 16.3 |
| 100 | 288.6 | 67.7 | 21.7 |
| 150 | 334.9 | 77.5 | 28.8 |
| 200 | 380.0 | 88.2 | 37.2 |
| 300 | 438.5 | 99.0 | 48.2 |

**Table S7** Comparison of microwave absorption performance for composite PCMs

| Sample | EAB  (GHz) | RL_min_  (dB) | Thickness  (mm) | Filler mass ratio (%) | Refs. |
| --- | --- | --- | --- | --- | --- |
| LA-CKF@Fe_3_O_4_ | 1.7 | -17.3 | 5.5 | 9.5% | S19 |
| PEG-CMF/rGO/MoS_2_ | 4.8 | -32.5 | 2.4 | 7.9% | S20 |
| PW-CMF@Co/NC | 3.8 | -57.9 | 3.0 | 39.8% | S21 |
| PW-MXene/CoNi-C | 3.1 | -49.3 | 4.5 | 28.0% | S22 |
| PW-MoS_2_@CNTs | 4.3 | -28.0 | 2.0 | 50.3% | S23 |
| PW-C-LDH@MXene | 3.9 | -20.9 | 2.0 | 30.4% | S24 |
| PEG-SWCNT/PDMS | 2.5 | -51.7 | 1.8 | 36.5% | S25 |
| PEG-PI/Graphene/Fe_3_O_4_ | 2.6 | -38.5 | 2.8 | 15.8% | S26 |
| PW-MXene/CNF@MoS_2_ | 4.3 | -61.3 | 1.4 | 33.5% | S27 |
| PW-Co-MnS/CF@MoS_2_ | 6.2 | -63.2 | 1.9 | 34.2% | S28 |
| PEG@FGA | 5.8 | 48.7 | 2.1 | 8.6% | S29 |
| ADGW/PEG | 4.9 | -41.4 | 2.5 | 16.7% | S30 |
| PI/PPy-CNTs@PEG | 2.6 | -42.0 | 5.5 | 10.8% | S31 |
| PEG-MXene@CuS | 5.3 | -55.5 | 1.9 | 33.0% | This work |

**Supplementary References**

[S1] S. J. Clark, M. D. Segall, C. J. Pickard, P. J. Hasnip, M. I. J. Probert, et al., First principles methods using castep. **220**(5–6), 567–570 (2005). https://doi.org/10.1524/zkri.220.5.567.65075

[S2] D. Vanderbilt, Soft self-consistent pseudopotentials in a generalized eigenvalue formalism. Phys. Rev. B **41**(11), 7892–7895 (1990). https://doi.org/10.1103/PhysRevB.41.7892

[S3] J. P. Perdew, K. Burke, M. Ernzerhof, Generalized gradient approximation made simple. Phys. Rev. Lett. **77**(18), 3865–3868 (1996). https://doi.org/10.1103/PhysRevLett.77.3865

[S4] S. Grimme, Semiempirical GGA‐type density functional constructed with a long‐range dispersion correction. J. Comput. Chem. **27**(15), 1787–1799 (2006). https://doi.org/10.1002/jcc.20495

[S5] H. J. Monkhorst, J. D. Pack, Special points for Brillouin-zone integrations. *Phys. Rev. B* **13**(12), 5188–5192 (1976). https://doi.org/10.1103/PhysRevB.13.5188

[S6] T. Parker, D. Zhang, D. Bugallo, K. Shevchuk, M. Downes, et al., Fourier-transform infrared spectral library of MXenes. Chem. Mater. **36**(17), 8437–8446 (2024). https://doi.org/10.1021/acs.chemmater.4c01536

[S7] S. Song, H. Ai, W. Zhu, L. Lv, R. Feng, et al., Carbon aerogel based composite phase change material derived from kapok fiber: Exceptional microwave absorbility and efficient solar/magnetic to thermal energy storage performance. Compos. Part B Eng. **226**, 109330 (2021). https://doi.org/10.1016/j.compositesb.2021.109330

[S8] Z. Hu, M. Jiang, Y. Zou, L. Sun, F. Xu, et al., MoS_2_-decorated carbonized melamine foam/reduced graphene oxide network for constructing polyethylene-glycol-based multifunctional phase change materials toward multiple energy harvesting and microwave absorbing applications. Chem. Eng. J. **461**, 141923 (2023). https://doi.org/10.1016/j.cej.2023.141923

[S9] Y. Li, X. Diao, P. Li, P. Liu, Y. Gao, et al., Advanced multifunctional Co/N co-doped carbon foam-based phase change materials for wearable thermal management. Chem. Eng. J. **485**, 149858 (2024). https://doi.org/10.1016/j.cej.2024.149858

[S10] Y. Gao, X. Chen, X. Jin, C. Zhang, X. Zhang, et al., Multifunction integration within magnetic CNT-bridged MXene/CoNi based phase change materials. eScience **4**(6), 100292 (2024). https://doi.org/10.1016/j.esci.2024.100292

[S11] P. Liu, Y. Li, Z. Tang, J. Lv, P. Cheng, et al., Integrating thermal energy storage and microwave absorption in phase change material-encapsulated core-sheath MoS_2_@CNTs. J. Energy Chem. **84**, 41–49 (2023). https://doi.org/10.1016/j.jechem.2023.04.048

[S12] Y. Gao, J. Lin, X. Chen, Z. Tang, G. Qin, et al., Engineering 2D MXene and LDH into 3D hollow framework for boosting photothermal energy storage and microwave absorption. Small **19**(49), 2303113 (2023). https://doi.org/10.1002/smll.202303113

[S13] X. Ge, G. Tay, Y. Hou, Y. Zhao, P. J. Sugumaran, et al., Flexible and leakage-proof phase change composite for microwave attenuation and thermal management. Carbon **210**, 118084 (2023). https://doi.org/10.1016/j.carbon.2023.118084

[S14] T. Shi, Z. Zheng, H. Liu, D. Wu, X. Wang, Configuration of multifunctional Polyimide/Graphene/Fe_3_O_4_ hybrid aerogel-based phase-change composite films for electromagnetic and infrared bi-stealth. Nanomaterials **11**(11), 3038 (2021). https://doi.org/10.3390/nano11113038

[S15] Y. Feng, G. Hai, G. Sun, K. Chen, X. Wang, et al., Dual-functional phase change composites integrating thermal buffering and electromagnetic wave absorption via multi-interfacial engineering. Adv. Fiber Mater. **7**(6), 1873–1887 (2025). https://doi.org/10.1007/s42765-025-00585-y

[S16] Y. Feng, M. Qin, J. Qin, Z. Shen, X. Chen, et al., Synergistic thermal management and electromagnetic wave absorption in core-sheath phase change composites. Adv. Funct. Mater. **36**(24), e23075 (2026). https://doi.org/10.1002/adfm.202523075

[S17] J. Zha, D. Feng, M. Zhang, X. Zhang, W. Li, et al., Graphene aerogel based/polyethylene glycol composite thermal storage materials with high enthalpy and low thermal conductivity for space thermal protection. J. Energy Storage **120**, 116456 (2025). https://doi.org/10.1016/j.est.2025.116456

[S18] L. Bai, S. Zhang, Z. Ma, L. Dou, J. Li, Bio-inspired multifunctional composite phase change films for synergistic thermal management and microwave absorption. Nano Res. **19**(5), 94908229 (2026). https://doi.org/10.26599/NR.2025.94908229

[S19] Y. Cao, Z. Zhao, X. Zeng, J. Teng, J. Huang, et al., High-performance Polyimide/Polypyrrole-CNTs@PEG composites for integrated thermal management and enhanced electromagnetic wave absorption. Adv. Compos. Hybrid Mater. **8**(1), 104 (2025). https://doi.org/10.1007/s42114-024-01202-z

[S20] Z. Dai, Y. Gao, C. Wang, D. Wu, Z. Jiang, et al., Oriented high thermal conductivity solid–solid phase change materials for mid-temperature solar-thermal energy storage. ACS Appl. Mater. Interfaces **15**(22), 26863–26871 (2023). https://doi.org/10.1021/acsami.3c04429

[S21] Y. Chen, Q. Ma, L. Chen, X. Wang, X. Zhao, et al., Enhanced light-to-thermal conversion performance of self-assembly carbon nanotube/graphene-interconnected phase change materials for thermal-electric device. J. Energy Storage **72**, 108387 (2023). https://doi.org/10.1016/j.est.2023.108387

[S22] G. Wu, N. Bing, Y. Li, H. Xie, W. Yu, Three-dimensional directional cellulose-based carbon aerogels composite phase change materials with enhanced broadband absorption for light-thermal-electric conversion. Energy Convers. Manage. **256**, 115361 (2022). https://doi.org/10.1016/j.enconman.2022.115361

[S23] H. Fang, Y. Heng, G. Teng, D. Hu, Advanced 3D-architected carbon-based photothermoelectric energy harvesters for self-powered and responsive magnetic and UV sensing. Ind. Crops Prod. **205**, 117483 (2023). https://doi.org/10.1016/j.indcrop.2023.117483

[S24] T. Shi, H. Liu, X. Wang, Unidirectionally structured magnetic phase-change composite based on carbonized Polyimide/Kevlar nanofiber complex aerogel for boosting solar-thermo-electric energy conversion. ACS Appl. Mater. Interfaces **16**(8), 10180–10195 (2024). https://doi.org/10.1021/acsami.3c18523

[S25] S. Han, F. Xiong, M. Qin, Z. Shen, H. Han, et al., Polyethylene glycol/polypyrrole aerogel shape-stabilized phase change material for solar-thermal energy storage and thermoelectric power generation. Sol. Energy Mater. Sol. Cells **268**, 112745 (2024). https://doi.org/10.1016/j.solmat.2024.112745

[S26] M. He, J. Lu, C. Shi, X. Qian, L. Yin, et al., Multi-stimuli-responsive aerogels composed of ag nanoparticle-coated graphene nanosheets for energy storage and conversion. ACS Appl. Nano Mater. **6**(18), 16503–16514 (2023). https://doi.org/10.1021/acsanm.3c02622

[S27] Y.-C. Zhou, J. Yang, L. Bai, R.-Y. Bao, M.-B. Yang, et al., Super-flexible phase change materials with a dual-supporting effect for solar thermoelectric conversion in the ocean environment. J. Mater. Chem. A **11**(1), 341–351 (2023). https://doi.org/10.1039/D2TA07885F

[S28] Q. Guo, H. Yi, F. Jia, S. Song, Novel MoS_2_/montmorillonite hybrid aerogel encapsulated PEG as composite phase change materials with superior solar-thermal energy harvesting and storage. J. Colloid Interface Sci. **667**, 269–281 (2024). https://doi.org/10.1016/j.jcis.2024.04.107

[S29] S. Li, N. Zhang, H. Chen, Z. Zhang, Y. Zhao, et al., Encapsulating phase change materials into melamine formaldehyde sponge assembled with polypyrrole modified halloysite nanotube for effective solar-thermal energy storage and solar-thermal-electric conversion. J. Colloid Interface Sci. **682**, 423–435 (2025). https://doi.org/10.1016/j.jcis.2024.11.226

[S30] Y. Li, X. Wang, K. Chen, Y. Feng, P. Liu, et al., Hierarchical MoS_2_/CuS photonic nanostructure accelerating photothermoelectric conversion of bacterial cellulose based phase change materials. J. Colloid Interface Sci. **702**, 138863 (2026). https://doi.org/10.1016/j.jcis.2025.138863

[S31] Y. Li, Y. Feng, M. Qin, K. Chen, Y. An, et al., Co-anchored hollow carbonized kapok fiber encapsulated phase change materials for upgrading photothermal utilization. Small **21**(21), 2500479 (2025). https://doi.org/10.1002/smll.202500479
